# Association of Blood Glucose Data With Physiological and Nutritional Data From Dietary Surveys and Wearable Devices: Database Analysis

**DOI:** 10.2196/62831

**Published:** 2024-12-03

**Authors:** Takashi Miyakoshi, Yoichi M Ito

**Affiliations:** 1 Department of Health Data Science Hokkaido University Graduate School of Medicine Sapporo Japan; 2 Data Science Center, Promotion Unit, Institute of Health Science Innovation for Medical Care Hokkaido University Hospital Sapporo Japan

**Keywords:** PhysioNet, Empatica, Dexcom, acceleration, heart rate, temperature, electrodermal activity

## Abstract

**Background:**

Wearable devices can simultaneously collect data on multiple items in real time and are used for disease detection, prediction, diagnosis, and treatment decision-making. Several factors, such as diet and exercise, influence blood glucose levels; however, the relationship between blood glucose and these factors has yet to be evaluated in real practice.

**Objective:**

This study aims to investigate the association of blood glucose data with various physiological index and nutritional values using wearable devices and dietary survey data from PhysioNet, a public database.

**Methods:**

Three analytical methods were used. First, the correlation of each physiological index was calculated and examined to determine whether their mean values or SDs affected the mean value or SD of blood glucose. To investigate the impact of each physiological indicator on blood glucose before and after the time of collection of blood glucose data, lag data were collected, and the correlation coefficient between blood glucose and each physiological indicator was calculated for each physiological index. Second, to examine the relationship between postprandial blood glucose rise and fall and physiological and dietary nutritional assessment indices, multiple regression analysis was performed on the relationship between the slope before and after the peak in postprandial glucose over time and physiological and dietary nutritional indices. Finally, as a supplementary analysis to the multiple regression analysis, a 1-way ANOVA was performed to compare the relationship between the upward and downward slopes of blood glucose and the groups above and below the median for each indicator.

**Results:**

The analysis revealed several indicators of interest: First, the correlation analysis of blood glucose and physiological indices indicated meaningful relationships: acceleration SD (*r*=–0.190 for lag data at –15-minute values), heart rate SD (*r*=–0.121 for lag data at –15-minute values), skin temperature SD (*r*=–0.121), and electrodermal activity SD (*r*=–0.237) for lag data at –15-minute values. Second, in multiple regression analysis, physiological indices (temperature mean: *t*=2.52, *P*=.01; acceleration SD: *t*=–2.06, *P*=.04; heart rate_30 SD: *t*=–2.12, *P*=.04; electrodermal activity_90 SD: *t*=1.97, *P*=.049) and nutritional indices (mean carbohydrate: *t*=6.53, *P*<.001; mean dietary fiber: *t*=–2.51, *P*=.01; mean sugar: *t*=–3.72, *P*<.001) were significant predictors. Finally, the results of the 1-way ANOVA corroborated the findings from the multiple regression analysis.

**Conclusions:**

Similar results were obtained from the 3 analyses, consistent with previous findings, and the relationship between blood glucose, diet, and physiological indices in the real world was examined. Data sharing facilitates the accessibility of wearable data and enables statistical analyses from various angles. This type of research is expected to be more common in the future.

## Introduction

### Global Prevalence of Diabetes and Hyperlipidemia

The global prevalence of diabetes in 2021 among individuals aged between 20 and 79 years was estimated to be 10.5% (536.6 million people), with this figure expected to rise to 12.2% (783.2 million) by 2045. Global diabetes-related health expenditures were estimated at US $966 billion in 2021 and are projected to reach US $1054 billion by 2045 [1]. In 2020, approximately 38.1 million adults aged ≥18 years, or 14.7% of all adults in the United States, had diabetes [2]. The global prevalence of hyperlipidemia in adults is 39% (37% in men and 40% in women), and as of 2020, approximately 10% (about 25 million people) of adults aged ≥20 years in the United States had total cholesterol levels of ≥240 mg/dL and 33% (about 86 million people) had total cholesterol levels of ≥200 mg/dL [3]. Several primary hyperlipidemias (eg, familial combined hyperlipidemia, familial hypertriglyceridemia, and abnormal β lipoproteinemia) have been reported to be associated with an increased risk of type 2 diabetes [4].

### The Role of Wearable Devices and Real-World Data

Wearable devices are becoming increasingly capable of measuring a range of physiological data. Real-world data, which collect and analyze activity and physiological measurements from participants in clinical studies, can provide more sensitive measures of disease activity than traditional scales, thereby enabling faster and more objective readings in clinical trials [5]. Wearable devices can continuously collect measures of multiple physiological functions in real-time. Depending on the size and complexity of the raw data obtained from the device, data preprocessing, feature extraction, and selection are performed through data processing, such as data mining, and are applied toward abnormality detection, prediction, diagnosis, and decision-making [6]. Conversely, data repositories have advanced in recent years, making large, open databases available for wearable devices. Data mining techniques have advanced significantly in the last few years, and with the availability of these open data, opportunities emerged to devise algorithms suitable for wearable sensors [6].

### Use of Open Datasets and Research Progress

Table S1 in Multimedia Appendix 1 depicts studies that applied existing open datasets. Several studies were conducted to establish algorithms and prediction models based on machine learning from sensor data [7-24].

Open datasets are available on the following websites: (1) IEEE Data Port, a research data platform designed to store research data and provide global access to research data across various fields [25]; (2) the Open Wearables Initiative, which aims to promote the effective use of high-quality sensor-generated health measurements in clinical research by openly sharing algorithms and datasets [26]; (3) PhysioNet, a searchable database containing a collection of cardiopulmonary, neurological, and other biomedical signals from healthy individuals and patients with several serious health conditions, including congestive heart failure, epilepsy, gait disturbance, and sleep apnea [27].

### Development of Prediction Models for Blood Glucose Indicators

The BIG IDEAs Lab Glycemic Variability and Wearable Device Data (version 1.1.1) by Cho et al [28,29], a wearable database containing blood glucose–related indicators from PhysioNet, was selected for the study. Two previous studies have used the same database [30,31]. The first study demonstrated the feasibility of predicting blood glucose changes by continuously detecting individualized blood glucose deviations and determining the contribution of each variable to interstitial glucose prediction. The LOPOCV random forest regression model was used to examine the importance of the characteristics, resulting in the extraction of *diet*, *circadian rhythm*, *stress*, *activity*, *body temperature*, *heart rate*, *electrodermal activity*, *biological sex*, and *HbA_1c_* [30]. The second study evaluated methods for detecting prediabetes and estimating glycated hemoglobin (HbA_1c_) and glucose variability using digital biomarkers from wearables [31]. The relationships between features extracted from wearables and blood glucose variability and HbA_1c_ were investigated, and the results showed that glucose variability indices and HbA_1c_ could be estimated with high accuracy. The HbA_1c_ estimation model developed from a noninvasive wrist-worn wearable was as accurate as the invasive continuous glucose monitor (CGM)–based estimated A_1c_ (as recommended by the American Diabetes Association). Notably, all the sensors used in this study (triaxial accelerometer-derived acceleration [ACC], heart rate [HR], electrodermal activity [EDA], and skin temperature [TEMP]) were important for estimating glucose variability indices and HbA_1c_, although EDA and TEMP were the most important indicators when estimating HbA_1c_ [31]. Similar to the other studies based on existing open datasets (Table S1 in Multimedia Appendix 1), these 2 studies were conducted to establish a prediction model for blood glucose-related indicators using machine learning and used the random forest regression model for analysis. This analysis can be difficult to interpret in a specific clinical context.

### Factors Influencing Blood Glucose Levels and Real-World Evaluation

Several factors influence blood glucose levels, including diet [32], physical activity, exercise [33], stress [34], circadian rhythm [35], and HR [36]. For example, regarding diet, following the dietary approaches to stop hypertension (DASH) diets [37], low carbohydrate diets [38,39], and high consumption of phytochemicals and polyphenols [40,41] prevent type 2 diabetes. However, although some factors associated with blood glucose variability, such as HR [36], body temperature [42], and autonomic functions [43], including sweating motor response [44], have been reported [30], these factors have not been evaluated in real-world setting. Therefore, we conducted an exploratory study of the association between blood glucose and each physiological and nutritional index, using existing data from PhysioNet as well as simple analytical methods, such as correlation and multiple regression analyses. These analysis methods were used for their simplicity compared to the random forest model and may increase the explanatory potential as the contribution of each variable is clarified.

## Methods

### Database Selection and Dataset Creation

A database search was conducted using the word “wearable” from PhysioNet, which yielded 11 hits. Among these, we focused on wristband-type wearable devices and searched the relevant databases because the major market share is dominated by wristband- and watch-type devices [45]. The search resulted in 7 (64%) of 11 studies with wristband-type wearable devices (Empatica [Empatica Inc]: n=5, 45%; Apple watch [Apple Inc]: n=2, 18%; Fitbit [Fitbit, Inc]: n=2, 18%; Garmin [Garmin Ltd]: n=1, 9%; Samsung Galaxy watch [Samsung, Inc]: n=1, 9%; Xiaomi [Xiaomi Corp]: n=1, 9%; and Biovotion Everion [Biofourmis Inc]: n=1, 9% study; Table S2 in Multimedia Appendix 1).

We selected 1 study that investigated Empatica E4, the most commonly used wristband-type wearable device. The dataset, BIG IDEAs Lab Glycemic Variability and Wearable Device Data (version 1.1.1) by Cho et al [28,29] was selected to examine the correlation between each physiological index and blood glucose as well as conduct multiple regression analysis and 1-way ANOVA of the slope in postprandial glucose over time with each physiological index and nutrient value. The main eligibility criteria in the study included men and postmenopausal women aged 35-65 years, with point-of-care HbA_1c_ measurements between 5.2% and 6.4%.

A total of 16 patients (n=7, 44% men and n=9, 56% women) with HbA_1c_ in the high normal and prediabetic range (5.3%-6.4%, mean 5.73%, SD 0.28%) were included and monitored for 8-10 days using the Dexcom G6 CGM and Empatica E4 wrist-worn wearable-type device [28].

The demographic characteristics of the participants are listed in Table 1.

**Table 1:** Demographic characteristics of the participants in the database.

ID	Sex	HbA_1c_^a^ (%)
a01	Female	5.5
a02	Male	5.6
a03	Female	5.9
a04	Female	6.4
a05	Female	5.7
a06	Female	5.8
a07	Female	5.3
a08	Female	5.6
a09	Male	6.1
a10	Female	6.0
a11	Male	6.0
a12	Male	5.6
a13	Male	5.7
a14	Male	5.5
a15	Female	5.5
a16	Male	5.5

^a^HbA_1c_: glycated hemoglobin.

Notably, all data were time-shifted (by date) to prevent reidentification; the Dexcom G6 measured interstitial glucose concentration (mg/dL) every 5 minutes using a CGM, and the Empatica E4 measured photoelectric volumetric pulse wave, electrical activity: EDA, TEMP, and ACC, for 7 functions. The photoelectric volumetric pulse wave was sampled at 64 Hz, and HR and blood volume pulse (BVP) signals were obtained every second, from which the interbeat interval (IBI) data were calculated. Of these, EDA is known as a psychological factor and a measure of sympathetic activation related to stress [46,47]. In addition, HR variability, a related index of HR and IBI, was used as a psychological stress indicator [47,48]. EDA and TEMP were sampled at 4 Hz, whereas accelerometry was sampled at 32 Hz. For ACC, triaxial data were calculated using the Euclidean norm as a measure of average motion in the 3 axes using the following formula [49]:



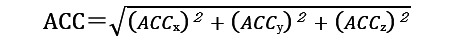



Each physiological index (ACC, HR, TEMP, EDA, BVP, and IBI) collected using Empatica was also extracted at 5-minute intervals to match blood glucose, which had the longest measurement interval (5 minutes).

In addition, when the Cho et al [28] dataset was updated from version 1.0.0 to version 1.1.0 on March 6, 2023, the results of nutrient value calculations from the dietary survey records were added to the analysis dataset. The parameters of the calculated nutritional values were calories, total carbohydrate (carbon), dietary fiber, sugar, protein, and total fat. The values of these nutritional assessment indices at each time point were summed.

### Correlation Analyses Between Blood Glucose and Each Physiological Index

The correlation of each physiological index (ACC, HR, TEMP, EDA, BVP, and IBI) was calculated and examined to determine whether their mean values or SDs affected the mean value or SD of blood glucose. Mean values and SDs were calculated for blood glucose and each physiological index at three 5-minute points in the same individual (10 minutes in total), and their correlation coefficients were calculated. To examine the impact of each physiological indicator on blood glucose before and after the time of collection of blood glucose data, lag data (data on physiological indicators before glucose data collection at 8 points [120, 105, 90, 75, 60, 45, 30, and 15 minutes] and after blood glucose data collection at 8 points [15, 30, 45, 60, 75, 90, 105, and 120 minutes]) were collected, and the correlation coefficient between blood glucose and each physiological indicator was calculated for each physiological index.

Lag data for physiological indicators before glucose data collection were created by time-shifting each physiological indicator every 15 minutes until 120 minutes (Figure S1 in Multimedia Appendix 1). Lag data for physiological index data after blood glucose data collection were time-shifted by 15 minutes for each glucose reading to 120 minutes (Figure S2 in Multimedia Appendix 1). The purpose of creating lag data was to calculate the correlation between glucose levels and ACC (ACC values at 15, 30, 45, 60, 75, 90, 105, and 120 minutes) before and after measurement. The correlation coefficient was calculated using Spearman correlation.

### Multiple Regression Analysis of Postprandial Blood Glucose Over Time and Each Physiological Index and Nutrient Value

To examine the relationship between postprandial blood glucose rise and fall and physiological and dietary nutritional assessment indices, multiple regression analysis was performed on the relationship between the slope before and after the peak in postprandial glucose over time and physiological and dietary nutritional indices. Multiple regression analysis was performed using objective and explanatory variables.

#### Objective Variables

The objective variables included the slope of the tangent line to the postprandial blood glucose curve, which includes the slope of the rise in postprandial glucose from the lowest point before the rise to the peak and the slope of postprandial blood glucose from the peak to the lowest point.

The following formula was used to calculate the slope:



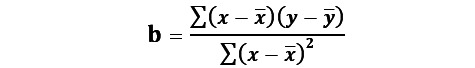



where x indicates time (min) and y indicates blood glucose (mg/dL).

#### Explanatory Variables

**Figure 1:**
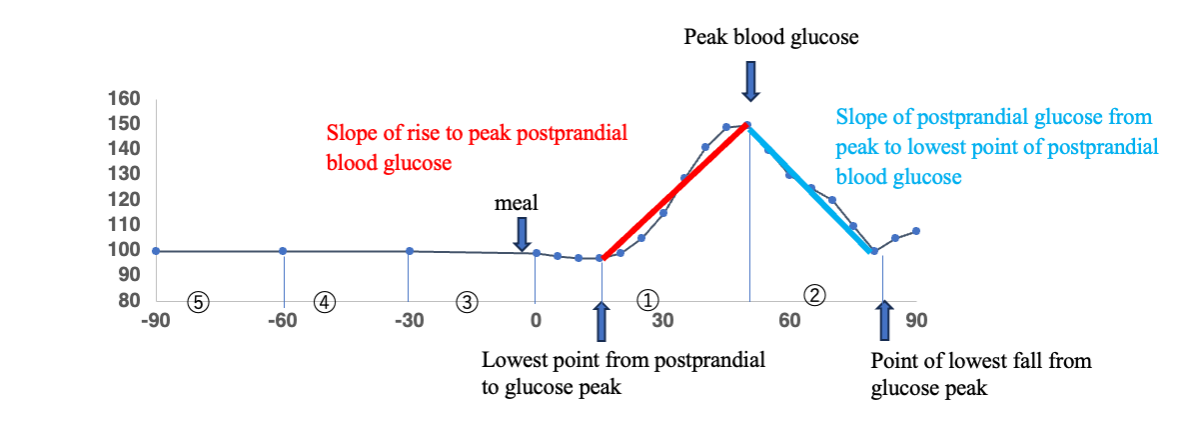
Collection times for the objective (blood glucose slope) and explanatory (physiological indexes) variables used in the multiple regression analysis.

The explanatory variables comprised the calculated nutritional value of the diet (carbon, protein, calories, sugar, dietary fiber, protein, and total fat) and the means and SDs of physiological indices (ACC, HR, TEMP, EDA, BVP, and IBI) at the following time points (Figure 1):

1. Time from the most recent postprandial glucose to peak glucose level. The variables are ACC, HR, TEMP, EDA, BVP, and IBI.
2. Time from peak glucose to the lowest glucose level (excluded from the analysis of the slope of peak ascent owing to limited data). The variables are ACC_af, HR_af, TEMP_af, EDA_af, BVP_af, and IBI_af.
3. Time from the most recent postprandial glucose peak to 30 minutes before the most recent postprandial glucose. The variables are ACC_30, HR_30, TEMP_30, EDA_30, BVP_30, and IBI_30.
4. Time from 30 minutes before the most recent postprandial glucose to 60 minutes before. Variables are ACC_60, HR_60, TEMP_60, EDA_60, BVP_60, and IBI_60.
5. Time from 60 minutes before the most recent postprandial glucose to 90 minutes before. Variables are ACC_90, HR_90, TEMP_90, EDA_90, BVP_90, and IBI_90.

Multiple regression analysis was performed using the variable reduction method, and the significance level used as the criterion for the backward elimination method [50,51] was *P*=.20.

### One-Way ANOVA for Postprandial Blood Glucose and Groups Above and Below Each Physiological Indicator and the Median Dietary Nutrient Value

As a supplementary analysis to the multiple regression analysis, a 1-way ANOVA was performed to compare the relationship between the upward and downward slopes of blood glucose and the groups above and below the median for each indicator. The groups below the median were used as comparison controls. The following variables were used in the 1-way ANOVA.

#### Objective Variables

The same variables were used for analysis as those in the multiple regression analysis.

#### Explanatory Variables

For the following indices, variables were created through patterns of group combinations using the physiological index mean and dietary nutrient value, with groups above and below the median for each index as 1 and 0, respectively. Group combinations that contained missing measures or extremely low group combinations were not included in the analysis population. The mean values of physiological indices (ACC, HR, TEMP, and EDA) at three 5-minute intervals (10 minutes in total) in the same participant at the following times and the nutritional value of the diet (carbon, protein, calories, sugar, and dietary fiber) were assessed.

The results of the multiple regression analysis showed that the physiological indicators associated with the slope of the rise and fall of blood glucose were TEMP, ACC, HR, and EDA, and the nutritional value indicators were carbon, protein, calories, sugar, and dietary fiber. Therefore, we focused on the following time indicators:

1. Time from the most recent postprandial glucose to peak glucose level.
2. Time from peak glucose to lowest glucose level (excluded from this analysis).
3. Time from the most recent postprandial glucose to 30 minutes before.
4. Time from 30 minutes before the most recent postprandial glucose to 60 minutes before.
5. Time from 60 minutes before the most recent postprandial glucose to 90 minutes before.

Patterned combination of groups by physiological indicator mean and dietary nutrient value are given in Textbox 1.

All analyses were performed using JMP Pro (version 16.10; SAS Institute Inc), SAS (version 9.4; SAS Institute Inc), and Microsoft Excel for Mac (version 16; Microsoft Corp).

**Patterned combination of groups.**

**Combination patterns of physiological index mean groups. Combination pattern of temperature (TEMP), acceleration (ACC), heart rate (HR), and electrodermal activity (EDA); for example,**

- Combination pattern for groups with TEMP, ACC, HR, and EDA below the median (used as a comparison). TEMP: ACC:HR:EDA=0000.
- Combination pattern for groups with TEMP, ACC, HR, and EDA above the median. TEMP: ACC:HR:EDA=1111.
- Combination pattern for groups where TEMP and EDA were above the median and all other values were below the median. TEMP:ACC:HR:EDA=1001.

**Combination patterns for dietary nutrient value groups; for example,**

- Combination pattern for all indicators of dietary nutritional value (used as comparisons and controls) below the median. Calories:carbon:dietary fiber:sugar:protein=00000.
- Combination pattern for all indicators of dietary nutritional value above the median. Calories:carbon:dietary fiber:sugar:protein=11111.
- Combinations pattern for carbon and sugar above the median and all other indicators of dietary nutritional value below the median. Calories:carbon:dietary fiber:sugar:protein=01010.

### Ethical Considerations

No ethical approval was required since this study was exclusively based on published literature.

## Results

### Correlation Coefficients Between Blood Glucose Mean and the Mean of Lag Data for Each Physiological Indicator

#### Lag Data for Each Physiological Indicator Before Blood Glucose Data Collection

**Figure 2:**
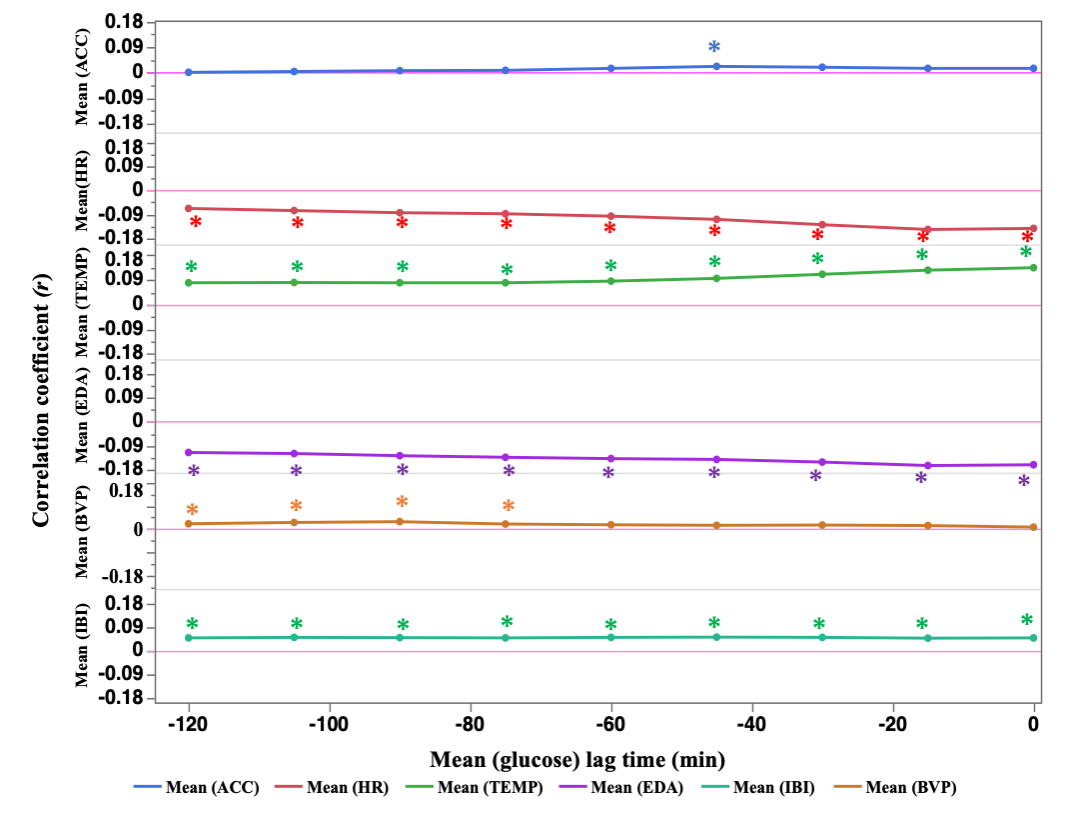
Trends in correlations between mean blood glucose and the mean of lag data for each physiological indicator (lag data for physiological indicators before blood glucose data collection). ACC: triaxial accelerometer–derived acceleration; BVP: blood volume pulse; EDA: electrodermal activity; HR: heart rate; IBI: interbeat interval; TEMP: skin temperature. *P value of correlation coefficient *P*<.05.

Figure 2 and Table S3 in Multimedia Appendix 1 illustrate the evolution of the correlation coefficients of the lag data (–120, –105, –90, –75, –60, –45, –30, and –15 min) between the mean values of blood glucose and physiological indices. For HR, the correlation remained negative, peaking at the –15-minute value of lag data (*r*=–0.147). For TEMP, the correlation remained positive, with a peak at the 0-minute value (*r*=0.135). For EDA, the correlation remained negative, peaking at the –15-minute value of lag data (*r*=–0.164).

#### Lag Data for Each Physiological Indicator After Blood Glucose Data Collection

Figure S3 and Table S4 in Multimedia Appendix 1 illustrate the evolution of the correlation coefficients of the lag data (15, 30, 45, 60, 75, 90, 105, and 120 min) for the mean values of glucose and physiological indices.

For HR, the correlation remained negative and peaked at the 45-minute value of lag data (*r*=–0.147). For TEMP, the correlation remained positive, peaking at the 60-minute value of lag data (*r*=0.161). For EDA, the correlation remained negative, with a peak at 0 minutes (*r*=–0.161). For IBI, the correlation remained positive, peaking at the 120-minute value of lag data (*r*=0.120).

### Correlation Coefficients Between Blood Glucose Mean and SD of Lag Data for Each Physiological Indicator

#### Lag Data for Each Physiological Indicator Before Blood Glucose Data Collection

**Figure 3:**
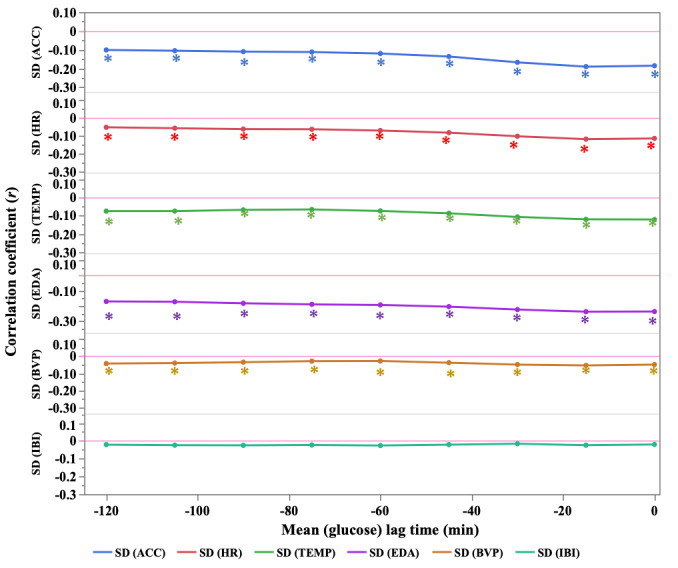
Trends in correlations between mean blood glucose values and SD of lag data for each physiological indicator (lag data for physiological indicators before blood glucose data collection). ACC: triaxial accelerometer derived acceleration; BVP: blood volume pulse; EDA: electrodermal activity; HR: heart rate; IBI: interbeat interval; TEMP: skin temperature. *P value of correlation coefficient *P*<.05.

Figure 3 and Table S5 in Multimedia Appendix 1 illustrate the evolution of the correlation coefficients of the lag data (–120, –105, –90, –75, –60, –45, –30, and –15 min) of the mean blood glucose values and SDs of physiological indices.

Regarding the evolution of the correlation coefficient of the lag data, which are the data at and after the time of blood glucose measurement, the correlation remained negative and peaked at the 15-minute value of lag data (*r*=–0.190) for ACC. For HR, the correlation remained negative, peaking at the 15-minute value of lag data (*r*=–0.121). For TEMP, the correlation remained negative, with a peak at the 0-minute value (*r*=–0.121). For EDA, the correlation remained negative, peaking at the 15-minute value of lag data (*r*=–0.237).

#### Lag Data for Each Physiological Indicator After Blood Glucose Data Collection

Figure S4 and Table S6 in Multimedia Appendix 1 illustrate the evolution of the correlation coefficients of the lag data (15, 30, 45, 60, 75, 90, 105, and 120 min) for the mean blood glucose values and SDs of physiological indices.

Regarding the evolution of the correlation coefficients of the lag data, which are the data at and after the time when blood glucose was measured, the correlation remained negative and peaked at the 0-minute value (*r*=–0.185) for ACC. For HR, the correlation remained negative, peaking at the 45-minute value of lag data (*r*=–0.127). For TEMP, the correlation remained negative, with a peak at 0-minute value (*r*=–0.121). For EDA, the correlation remained negative, with a peak at the 0-minute value (*r*=–0.236).

### Correlation Coefficients Between SD of Blood Glucose and SD of Lag Data for Each Physiological Indicator

#### Lag Data for Each Physiological Indicator Before Blood Glucose Data Collection

Figure S5 and Table S7 in Multimedia Appendix 1 illustrate the evolution of the correlation coefficients of the lag data (–120, –105, –90, –75, –60, –45, –30, and –15 min) for the SD of blood glucose and physiological indices.

Regarding the evolution of the correlation coefficients of the lag data, which are the data at and after the time of glucose measurement, the correlation remained positive, with a peak at the 0-minute value for ACC, HR, and TEMP (*r*=0.157, *r*=0.142, and *r*=0.127, respectively).

#### Lag Data for Each Physiological Indicator After Blood Glucose Data Collection

Figure S6 and Table S8 in Multimedia Appendix 1 illustrate the evolution of the correlation coefficients of the lag data (15, 30, 45, 60, 75, 90, 105, and 120 min) for the SD of glucose and physiological indices.

For the transition of the correlation coefficients for the lag data, which are the data at and after the time of glucose measurement, the correlation remained positive and peaked at the 0-minute value (*r*=0.157) for ACC. For HR, the correlation remained positive, with a peak at the 0-minute lag data (*r*=0.142). For TEMP, the correlation remained positive, with a peak at the 0-minute value (*r*=0.127).

### Results of Regression Analysis of Postprandial Glucose Over Time and Each Physiological Index and Nutrient Value

#### Results of Multiple Regression Analysis Between the Slope of the Peak of Blood Glucose Rise and the Mean Values of Physiological and Nutritional Assessment Indices

The results of multiple regression analysis between the slopes of the elevated blood glucose peak and the mean values of physiological and nutritional assessment indices are shown in Table S9 and S10 in Multimedia Appendix 1.

The physiological index (TEMP: *t*=2.52, *P*=.01) and nutritional assessment indexes (calorie: *t*=–3.98, *P*<.001, carbon: *t*=6.53; *P*<.001, dietary fiber: *t*=–2.51, *P*=.01, and protein: *t*=3. 82, *P*<.001) suggest that the mean values of these nutritional measures were significantly associated with the slope of the peak blood glucose elevation.

#### Results of Multiple Regression Analysis Between the Slope of the Descending Peak of Blood Glucose and the Mean Values of Physiological and Nutritional Assessment Indexes

The results of multiple regression analysis between the slope of the blood glucose descending peak and the mean values of physiological and nutritional assessment indices are shown in Table S11 and S12 in Multimedia Appendix 1.

The physiological indices (ACC: *t*=2.67, *P*=.008, HR_af: *t*=3.86; *P*<.001, and HR_90: *t*=2.27, *P*=.02), and nutritional assessment index (sugar: *t*=–3.72, *P*<.001) suggest that the mean values of these physiological and nutritional assessment measures were significantly associated with the slope of the descending peak of blood glucose.

#### Results of Multiple Regression Analysis Between the Slope of the Peak of Blood Glucose Rise and SD of Physiological and Nutritional Assessment Indexes

The results of multiple regression analysis between the slope of the peak of elevated blood glucose and the SD of physiological indices are shown in Tables S13 and S14 in Multimedia Appendix 1.

The physiological indices (ACC: *t*=–2.06, *P*=.04, HR_30: *t*=–2.12, *P*=.03, and EDA_90: *t*=1.97, *P*=.049) suggest that the SD of these physiological indicators was significantly associated with the slope of the peak glucose rise.

#### Results of Multiple Regression Analysis Between the Slope of the Descending Peak of Blood Glucose and SD of Physiological and Nutritional Assessment Indexes

The results of multiple regression analysis between the slope of the blood glucose descending peak and SD of physiological and nutritional assessment indices are shown in Tables S15 and S16 in Multimedia Appendix 1.

None of the physiological indices showed a significant association between the SD of the physiological index and the slope of the blood glucose elevation peak.

Age [52], weight, BMI, blood lipid levels, blood pressure [53,54] and sex [55] affect blood glucose levels. However, since we obtained data from a public database, background information other than sex could not be obtained. When sex was added to the multiple regression analysis as an adjustment factor, the results were largely consistent with the unadjusted results (Tables S17-S24 in Multimedia Appendix 1).

#### Results of the 1-Way ANOVA of Postprandial Blood Glucose Over Time and Groups Above and Below Each Physiological Indicator and the Median Dietary Nutrient Value

##### Results of the 1-Way ANOVA of the Slope of Elevated Postprandial Blood Glucose and the Combined Pattern for Groups Above and Below the Median of the Mean of Each Physiological Index (TEMP, ACC, HR, and EDA)

**Figure 4:**
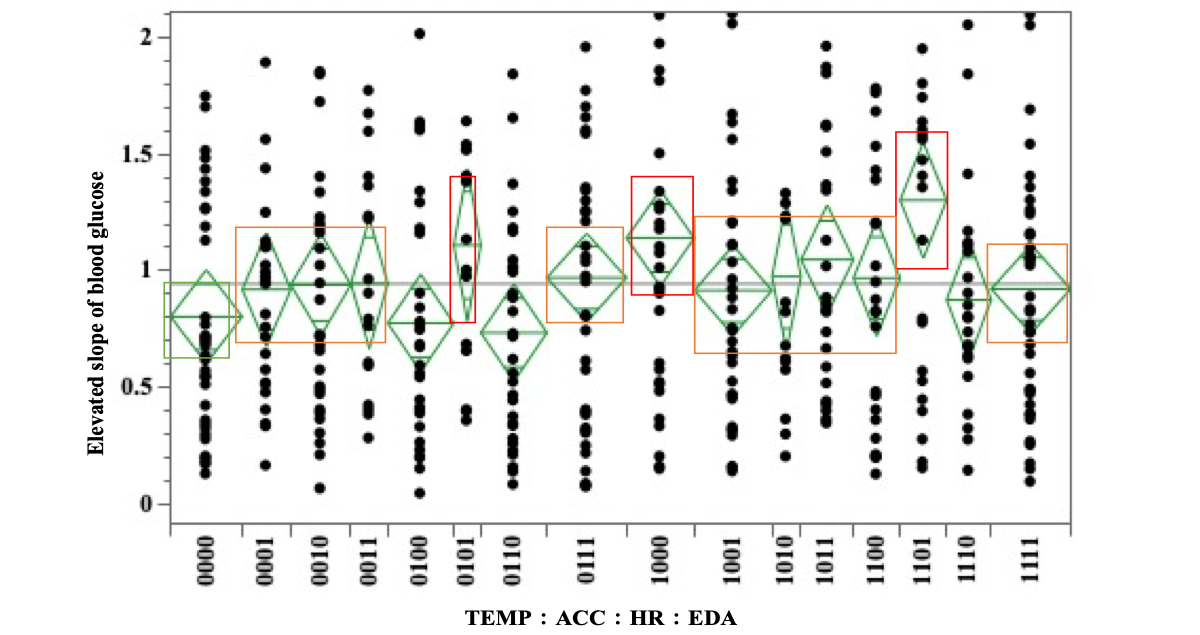
Relationship between the slope of blood glucose rise and the combination patterns (skin temperature [TEMP], triaxial accelerometer–derived acceleration [ACC], heart rate [HR], and electrodermal activity [EDA]).

The results of the 1-way ANOVA of the slope of elevated blood glucose and the combined pattern for the mean values of physiological indicators (TEMP, ACC, HR, and EDA) are shown in Figure 4 and Tables S25 and S26 in Multimedia Appendix 1.

The combination patterns of the group with a greater upward slope of blood glucose than the group with TEMP, ACC, HR, and EDA values all below the median (TEMP:ACC:HR:EDA=0000; mean value of upward slope 0.803) were TEMP:ACC:HR:EDA=0101 (mean value of upward slope 1.110), 1000 (mean value of upward slope 1.140), 1011 (mean value of upward slope 1.048), and 1101 (mean value of upward slope 1.303), with a larger upward slope in the population with higher TEMP and EDA.

Combination patterns with slightly larger values were TEMP:ACC:HR:EDA=0001 (mean value of upward slope 0.920), 0010 (mean value of upward slope 0.940), 0011 (mean value of upward slope 0.946), 0111 (mean value of upward slope 0.970), 1001 (mean value of upward slope 0.916), 1010 (mean value of upward slope 0.975), 1100 (mean value of upward slope 0.969), and 1111 (mean value of upward slope 0.922), which were also similar to the larger combination pattern groups.

##### Results of the 1-Way ANOVA of the Combined Pattern for Groups With the Downward Slope of Postprandial Blood Glucose and Higher and Lower Than Median Values of Each Physiological Index Mean

The results of the 1-way ANOVA of the combined pattern of the descending slope of blood glucose and mean physiological indices (TEMP, ACC, HR, and EDA) are shown in Figure S7 and Tables S27 and S28 in Multimedia Appendix 1.

The combination pattern with a greater downward slope of blood glucose than the group with TEMP, ACC, HR, and EDA values all below the median (TEMP:ACC:HR:EDA=0000, mean value of downward slope –0.663) was TEMP:ACC:HR:EDA=1011 (mean value of downward slope –1.045), with a greater downward slope in the population with higher TEMP, HR, and EDA. Combination patterns with slightly larger values were TEMP:ACC:HR:EDA=0111 (mean value of downward slope –0.751), 1000 (mean value of downward slope –0.752), 1010 (mean value of downward slope –0.785), 1100 (mean value of downward slope –0.787), and 1110 (mean value of downward slope –0.713), with the pattern combining HR and ACC with TEMP and EDA having a higher downward slope.

##### Results of the 1-Way ANOVA of the Slope of the Postprandial Rise in Blood Glucose and the Combined Pattern for Groups Above and Below the Median for Each Dietary Nutrient Value

**Figure 5:**
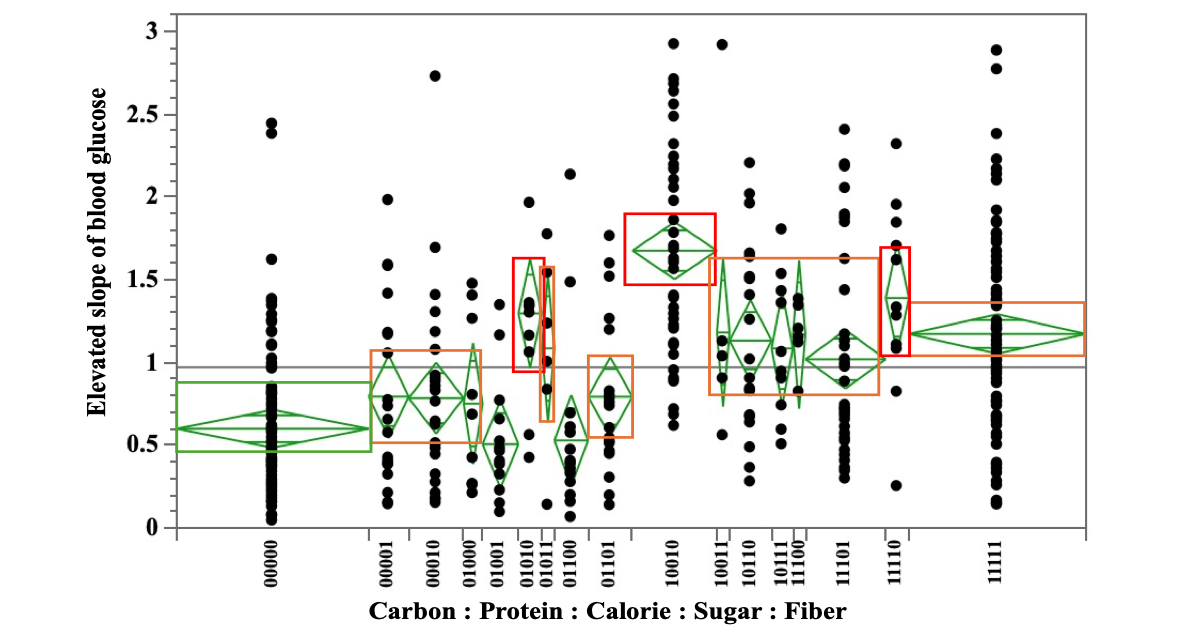
Relationship between the slope of the rise in blood glucose and dietary nutrient combination patterns (carbon, protein, calories, sugar, and fiber).

The results of the 1-Way ANOVA of the combined pattern of the upward slope of blood glucose and dietary nutrient values (carbon, protein, calories, sugar, and fiber) are shown in Figure 5 and Tables S29 and S30 in Multimedia Appendix 1.

A slightly smaller upward slope of blood glucose was observed than the group where carbon, protein, calories, sugar, and fiber were all below the median (carbon:protein:calories:sugar:fiber=00000; the mean value of upward slope 0.599), for the combination pattern of carbon:protein:calorie:sugar:fiber=01001 (mean value of the upward slope 0.506), which was a combination of protein and fiber.

The larger combination patterns were carbon:protein:calorie:sugar:fiber=01010 (mean value of upward slope 1.296), 11110 (mean value of upward slope 1.389), and 10010 (mean value of upward slope 1.675), with high calories and sugar, and the upward slope of the low fiber group was large.

Slightly larger combination patterns were observed for carbon:protein:calories:sugar:fiber=00001 (mean value of upward slope 0.751), 00010 (mean value of upward slope 0.784), 01101 (mean value of upward slope 0.792), 00001 (mean value of upward slope 0.793), 11101 (mean value of upward slope 1.019), 10111 (mean value of upward slope 1.084), 01011 (mean value of upward slope 1.084), 10110 (mean value of upward slope 1.132), 11100 (mean value of upward slope 1.168), 11111 (mean value of upward slope 1.172), and 10011 (mean value of the upward slope 1.181). Compared with the combination of carbon and sugar above the median, the combination of carbon and sugar above the median and fiber above the median had a smaller upward slope (carbon:protein:calorie:sugar:fiber=01010 [mean value of upward slope 1.296] and 01011 [mean value of upward slope 1.084]), 11110 [mean value of upward slope; 1.389] and 11111 [mean value of upward slope 1.172], 10010 [mean value of upward slope 1.675] and 10011 [mean value of upward slope 1.181]).

##### Results of the 1-Way ANOVA of the Downward Slope of Postprandial Blood Glucose and the Combined Pattern for Groups Above and Below the Median for Each Dietary Nutrient Value

The results of the 1-way ANOVA of the combined pattern of the downward slope of blood glucose and dietary nutrient values (carbon, protein, calories, sugar, and fiber) are shown in Figure S8 and Tables S31 and S32 in Multimedia Appendix 1.

The combination pattern of carbon:protein:calories:sugar:fiber=01100 (mean value of the downward slope –0.408), which was a combination of protein and calories, showed a slightly smaller downward slope in blood glucose than the group where carbon, protein, calorie, sugar, and fiber were all below the median (carbon:protein:calories:sugar:fiber=00000; mean value of the downward slope –0.525).

The larger combination patterns were carbon:protein:calories:sugar:fiber=01010 (mean value of the downward slope –1.012), 10111 (mean value of the downward slope –0.978), and 10010 (mean value of the downward slope –1.028). The slightly larger combination patterns were carbon:protein:calories:sugar:fiber=01011 (mean value of downward slope –0.626), 11101 (mean value of downward slope –0.762), 11100 (mean value of downward slope –0.769) and 11110 (mean value of downward slope –0.783), 00010 (mean value of downward slope –0.793), 10110 (mean value of downward slope –0.826) and 10011 (mean value of downward slope –0.884), than those with carbon and sugar above the median. Compared with the combination of carbon and sugar higher than the median, the combination of carbon and sugar higher than the median and fiber or protein higher than the median had a smaller downward slope (carbon:protein:calorie:sugar:fiber=01010 [mean of downward slope –1.012] and 01011 [mean of upward slope –0.626], 10111 [mean of upward slope –0.978] and 11111 [mean of upward slope –0.769], 10010 [mean value of upward slope –1.028] and 10011 [mean value of upward slope –0.884]).

## Discussion

### Correlation Analyses Between the Mean and SD of Glucose and the Mean of Each Physiological Index

We reviewed the evolution of the correlation coefficients, including the lag data for glucose, to determine whether the impact of each physiological indicator on glucose was influenced by physiological indicators before and after the time the glucose data were collected (Table 2).

The results showed that some indices such as ACC, TEMP, EDA, and IBI showed data correlations before and after the collection of blood glucose data. Activity, exercise, and stress are factors that influence blood glucose levels. Factors such as HR, body temperature, and autonomic nervous system function, including the sweating motor response, were associated with blood glucose variability [30]. The mean blood glucose and ACC SDs were negatively correlated; however, the uptake of blood glucose by mild to moderate physical activity is reported to cause decreased blood glucose levels and increased glucose production in the liver, leading to increased blood glucose [56]. This may be attributed to fluctuations in physical activity. The mean values of blood glucose and HR were negatively correlated; however, previous reports have indicated a negative relationship between blood glucose and HR variability, as sympathetic dominance increases with increasing blood glucose [57,58], which contradicts previous results. This could be attributed to, among others, increased in HR due to fasting-related hypoglycemia [58] and mild to moderate physical activity [56].

Regarding the correlation between mean blood glucose and mean TEMP, intravenous administration of blood glucose increased heat production by 20%, accompanied by an increase in TEMP after 55 minutes, which was presumed to be caused by this effect [59]. Regarding the correlation between blood glucose and EDA, EDA can increase during stress and is mediated by stress-induced activation of adrenergic hormones and cortisol. This increases blood glucose production and is thus positively related. Blood glucose levels increase in some individuals and decrease in others in response to stressful situations. Naturally occurring daily stressors may be associated with increased glycemic instability from hypoglycemia and decreased food intake, possibly due to these factors [60].

The mean blood glucose and SD of the physiological indices were negatively correlated.

This is because the mean and SD of physiological indicators were calculated from the mean and SD of the 3 points at individual 5-minute intervals, and the variation in physiological indicators was greater before and after the peak rise in glucose, whereas the variation in relevant physiological indicators was smaller at the peak of the rise in blood glucose. Meanwhile, the SD of blood glucose and physiological indicators were positively correlated, which was attributed to the increased variability in the SD of physiological indicators and blood glucose at times of blood glucose fluctuation (prepeak and peak transition).

**Table 2:** Summary of correlations between blood glucose and physiological indexes.

				*r*
**Correlation coefficients between mean values of blood glucose and mean values of physiological indices**				
	**Before blood glucose data collection**			
		HR^a^: negative correlation, peaking at –15 minutes	–0.147	
		TEMP^b^: positive correlation, peaking at 0 minutes	0.135	
		EDA^c^: negative correlation, peaking at 15 minutes	–0.164	
	**After blood glucose data collection**			
		HR: negative correlation, peaking at 45 minutes	–0.147	
		TEMP: positive correlation, peaking at 60 minutes	0.161	
		EDA: negative correlation, peaking at 0 minutes	–0.161	
		IBI^d^: positive correlation, peaking at 120 minutes	0.120	
**Correlation coefficients between mean values of blood glucose and SDs of physiological indices**				
	**Before blood glucose data collection**			
		ACC: negative correlation, peaking at 15 minutes	–0.190	
		HR: negative correlation, peaking at 15 minutes	–0.121	
		TEMP: negative correlation, peaking at 0 minutes	–0.121	
		EDA: negative correlation with EDA, peaking at 15 minutes	–0.237	
	**After blood glucose data collection**			
		ACC: negative correlation, peaking at 0 minutes	–0.185	
		HR: negative correlation, peaking at 45 minutes	–0.127	
		TEMP: negative correlation, peaking at 0 minutes	–0.121	
		EDA: negative correlation, peaking at 0 minutes	–0.236	
**Correlation coefficients between SD of blood glucose and SD of physiological indices**				
	**Before blood glucose data collection**			
		ACC: positive correlation, peaking at 0 minutes	0.157	
		HR: positive correlation, peaking at 0 minutes	0.142	
		TEMP: positive correlation, peak at 0 minutes	0.127	
	**After blood glucose data collection**			
		ACC: positive correlation, peaking at 0 minutes	0.157	
		HR: positive correlation, peaking at 0 minutes	0.142	
		TEMP: positive correlation, peak at 0 minutes	0.127	

^a^HR: heart rate.

^b^TEMP: skin temperature.

^c^EDA: electrodermal activity.

^d^IBI: interbeat interval.

### Slopes of the Rise to the Peak and Fall After the Peak in Blood Glucose Over Time After a Meal and the Results of the Regression Analysis of the Mean and SD of Each Physiological Index and Nutrient Value

To investigate the relationship between the slopes of the blood glucose rise and fall peaks and the mean values of physiological indices and nutritional assessment indices, as well as the relationship between the slope of the blood glucose rise and fall peaks and the mean values of physiological indices, a multiple regression analysis was conducted. A summary of the results is shown in Table 3.

**Table 3:** Summary of the relationship between the slopes of the blood glucose rise and fall peaks and the mean values of physiological and nutritional assessment indices as well as the relationship between the slope of the blood glucose rise and fall peaks and the mean values of physiological indices.

				Slope of peak blood glucose increase					Slope of blood peak glucose decrease		
				*t* value		*P* value		*t* value			*P* value
**Results of multiple regression analysis between the slope of the glucose peak and the mean values of physiological indices and nutritional assessment indices**											
	**Physiological indices**										
		Temperature	2.52		.01		—^a^			—	
		ACC^b^	—		—		2.67			.008	
		HR_af^c^	—		—		3.86			<.001	
		HR_90^d^	—		—		2.27			.02	
	**Nutritional assessment indices**										
		Calorie	3.98		<.001		—			—	
		Carbon	6.53		<.001		—			—	
		Dietary fiber	2.51		.01		—			—	
		Protein	3.82		<.001		—			—	
		Sugar	—		—		–3.72			<.001	
**Results of multiple regression analysis between the slope of the glucose peak and the SD of physiological indices**											
	**Physiological indices**										
		ACC	2.06		.04		—			—	
		HR_30^e^	2.12		.03		—			—	
		EDA_90^f^	1.97		.049		—			—	

^a^Not available.

^b^ACC: triaxial accelerometer-derived acceleration.

^c^HR_af: heart rate_af.

^d^HR_90: heart rate_90.

^e^HR_30 heart rate_30.

^f^EDA_90: electrodermal activity_90.

The physiological and nutritional assessment indices associated with the slope of the peak blood glucose increase were TEMP, calories, carbon, dietary fiber, protein, ACC, HR_30, and EDA_90. For TEMP, a positive association was observed; however, this was presumably due to the reported association between increased glucose and TEMP [59]. For carbon, a positive association was found, which was thought to be because carbohydrates contribute to the increase in blood glucose, although they are not absorbed as rapidly as glucose [61].

Although the upward slope of blood glucose and the calorie mean were negatively associated, the 1-way ANOVA revealed a positive association, as did carbon. Therefore, this result may be due to the multicollinearity effect of simultaneously introducing carbon and calories (*r*=0.78; *P*<.001), which are strongly correlated as explanatory variables.

Dietary fiber intake lowers postprandial and average daily blood glucose levels [62]. Protein intake does not increase plasma glucose levels but rather decreases them; thus, protein intake with glucose suppresses the postprandial increase in glucose [63], contrasts with the expected result. This was inferred to be due to increased carbohydrate intake since total carbon and protein intake were positively correlated (*r*=0.49; *P*<.001). A negative association was found for ACC and HR, which was presumed to be physical activity-related increased HR, accompanied by decreased blood glucose levels [56]. A positive relationship was found for EDA_90. EDA increases during stress and is mediated by stress-induced activation of adrenergic hormones and cortisol, which increases gluconeogenesis [60].

The physiological and nutritional assessment indices associated with the slope of the descending glucose peak were ACC, sugar, HR_af, and HR_90; positive associations were found between the downward slope of blood glucose and ACC and HR mean values. The decrease in blood glucose is potentially due to an increase from mild to moderate physical activity (HR also increased with physical activity), resulting in a smaller upward slope of blood glucose and therefore creating a smaller downward slope [56]. Although a negative relationship was observed between the downward slope of blood glucose and sugar, similar to carbohydrates, sugar intake has been reported to increase blood glucose [64], The upward slope of blood glucose is greater when carbohydrate intake is higher, and therefore the downward slope is also greater.

### A 1-Way ANOVA for Groups With Higher and Lower Than Median Blood Glucose Rise and Fall Slopes and Mean Values of Each Physiological Indicator and Dietary Nutrient Value

As a supplementary analysis to the multiple regression analysis, a 1-way ANOVA was performed to compare the relationship between the upward and downward slopes of blood glucose and the groups above and below the median for each indicator, using variables created by patterned group combinations by physiological indicator mean and dietary nutrient value. In addition, below-median groups were used as comparison controls. A summary of the results is presented in Table S33.

The results of the multiple regression analysis showed that the physiological indicators associated with the slope of rising and falling blood glucose were TEMP, ACC, HR, and EDA, whereas the nutritional indicators were carbon, protein, calories, sugar, and dietary fiber; therefore, these indicators were of interest.

The combination pattern of physiological indicators TEMP:ACC:HR:EDA, which included TEMP and EDA, showed that the upward and downward slopes of blood glucose were greater in the group above than in the group below the median. The effect on the upward slope of blood glucose was particularly large, similar to the results of multiple regression analysis. The combination pattern TEMP:ACC:HR:EDA=1011, which had a large upward slope of blood glucose, was due to stress, with high EDA and HR. The combination patterns TEMP:ACC:HR:EDA=1101 and 1111 also reported high glucose levels during and after moderate-intensity exercise [65] and high EDA due to exercise-induced sweating [66]. TEMP and EDA are strongly associated with autonomic nervous system function, which, in turn, is sensitive to glucose fluctuations, especially hyperglycemia. Therefore, we inferred that a high association exists between these indicators and the increase and fall in blood glucose slopes [31].

Conversely, for ACC and HR, the upward and downward slopes of blood glucose were slightly smaller in the group above than in the group below the median, accompanied by decreased blood glucose levels due to mild to moderate increases in physical activity [56] and physical activity-related increased HR similar to the results of the multiple regression analysis.

In the combination pattern of the nutrient indices carbon:protein:calories:sugar:fiber, the combination pattern including carbon, calories, and sugar showed a greater upward and downward slope of blood glucose in the group above than in the group below the median.

Dietary fiber reduces postprandial and mean daily blood glucose levels [62]; however, a group pattern of combinations with a greater rise and fall slope in blood glucose levels exists, with fiber above the median. The rise and fall slopes were smaller than those for groups of combinations below the median, similar to the results of the multiple regression analysis.

### Previous Analyses

Two previous studies have used the wearable database in this study, BIG IDEAs Lab Blood Glycemic Variability and Wearable Device Data [30,31]. These studies were conducted to establish a prediction model for blood glucose-related indicators using machine learning and used a random forest regression model as the method of analysis. This analysis can be difficult to interpret in a specific clinical context.

In contrast, this study used simple analysis methods, such as correlation and multiple regression analyses, to explore the relationship between blood glucose and each physiological and nutritional index in a real-world setting. Despite the difference in analysis methods, the results for glucose and related indicators of our study were consistent with those of previous studies, reinforcing the results of this study. In addition, compared with the random forest model, these simpler analyses revealed more details of the relationship between blood glucose and each physiological and nutritional index.

Other publicly available open datasets on diabetes are shown in Table S1 in Multimedia Appendix 1. In total, 4 studies were conducted using PhysioNet’s D1NAMO Multimodal Dataset for Noninvasive Type 1 Diabetes Management Studies (2018) dataset. This dataset was collected to contribute to the development of data-centric algorithms and diabetes monitoring techniques by providing an openly available multimodal dataset. It was obtained from real patients in a nonclinical setting, containing electrocardiogram signals, respiratory signals, accelerometer output, blood glucose level information, and annotated food photographs [67]. Studies conducted with this dataset include the following: a study that used machine learning to predict blood glucose in patients with type 1 diabetes [20]; 1 study aimed at improving the accuracy of CGM systems [21]; an insulin absorption simulation study [22]; and a study for predicting diabetes [24], which is useful in several approaches.

This study has some limitations. First, as we used publicly available data, other data, such as detailed patient background data (height and weight) collected during a clinical study but not made publicly available, were not included in the analysis. Second, Empatica, a wearable device, continuously collects a vast amount of data on physiological indicators, whereas the Dexcom G6, which measures glucose, collects data at 5-minute intervals. For the analysis of the physiological indicators, data were extracted according to the glucose measurement interval, and data that were not extracted could not be considered. Third, as the data were from 16 participants, which is a small sample size, the calculation of correlation coefficients and multiple regression analysis were conducted; however, only exploratory studies were possible. Fourth, while data at the beginning of the meal were available for all cases, data at the end of the meal were available only for some cases. Therefore, in analyzing the relationship between the upward and downward slopes of postprandial glucose to the peak and the nutritional index of the meal, the nutritional index immediately before the peak was added together. Therefore, the effect of mealtime length may not have been considered.

### Conclusions

Existing data from clinical studies on wearable-type devices (Dexcom 6 CGM and Empatica) from PhysioNet, a public open dataset, were used secondarily to examine the association of blood glucose with physiological and nutritional indices in 16 patients with borderline diabetes. The results showed that physiological indices associated with blood glucose were physical activity, HR, TEMP, and EDA, a stress indicator. In addition, physiological indices that were associated with the slope of the peak of the rise and fall of blood glucose were TEMP, physical activity, HR, and EDA. Nutritional measures associated with the slope of the peak rise and fall of blood glucose were carbohydrates, dietary fiber, and sugars. For the 3 analyses, the physiological measures associated with blood glucose were similar and consistent with previous reports.

The wearable-type device dataset allowed for the examination of the relationship of blood glucose with physiological and nutritional indicators. Research using existing data is expected to increase as open datasets of wearable device data become more readily accessible through data sharing and as it becomes possible to perform statistical analysis from various angles using such data.

## Appendix

Multimedia Appendix 1 Supplementary tables and figures.

## Abbreviations

ACC: triaxial accelerometer-derived acceleration
BVP: blood volume pulse
CGM: continuous glucose monitor
DASH: dietary approaches to stop hypertension
EDA: electrodermal activity
HbA_1c_: glycated hemoglobin
HR: heart rate
IBI: interbeat interval
TEMP: skin temperature
