# Supplementary material for: Association of Blood Glucose Data With Physiological and Nutritional Data From Dietary Surveys and Wearable Devices: Database Analysis
Source: JMIR Diabetes. 2024 Dec 3;9:e62831. doi: 10.2196/62831 (PMC11653050; doi:10.2196/62831)
Supplement: Multimedia Appendix 1 [file diabetes_v9i1e62831_app1.docx]

**Table of contents**

**Supplementary Figure S1.** Lag data before data collection time shift at eight time points (**−**120, **−**105, **−**90, **−**75, **−**60, **−**45, **−**30, and **−**15 min); ACC: tri-axial accelerometer derived acceleration

**Supplementary Figure S2.** Lag data after data collection time shift at eight time points

(120, 105, 90, 75, 60, 45, 30, and 15 min)

**Supplementary Figure S3.** Trends in the correlations between mean blood glucose values and mean of lag data for each physiological indicator (lag data for physiological indicators after blood glucose data collection).

**Supplementary Figure S4.** Trends in the correlations between mean blood glucose values and lag data for each physiological indicator standard deviation (lag data for physiological indicators after blood glucose data collection).

**Supplementary Figure S5.** Trends in the correlations between standard deviations of blood glucose values and standard deviations of lag data for each physiological indicator (lag data for physiological indicators before blood glucose data collection).

**Supplementary Figure S6.** Trends in the correlations between standard deviations of blood glucose values and standard deviations of lag data for each physiological indicator (lag data for physiological indicators after blood glucose data collection).

**Supplementary Figure S7.** Relationship between the downward slope of blood glucose and the combination patterns (skin temperature [TEMP], tri-axial accelerometer derived acceleration [ACC], heart rate [HR], and electrodermal activity [EDA]).

**Supplementary Figure S8.** Relationship between the downward slope of blood glucose and the combination patterns (carbon, protein, calories, sugar, and fiber).

**Supplementary Table S1.** Studies using existing open datasets

**Supplementary Table S2.** Outcomes of the search for a database of wearable-type device research database in PhysioNet.

**Supplementary Table S3.** Trends in the correlations between mean blood glucose and the mean of lag data for each physiological indicator (lag data for physiological indicators before blood glucose data collection)

**Supplementary Table S4.** Trends in the correlations between mean blood glucose and the mean of lag data for each physiological indicator mean (lag data for physiological indicators since blood glucose data collection)

**Supplementary Table S5.** Trends in thecorrelations between mean blood glucose and standard deviation of lag data for each physiological indicator (lag data for physiological indicators before blood glucose data collection)

**Supplementary Table S6.** Trends in the correlations between mean blood glucose and standard deviation of lag data for each physiological indicator (lag data for physiological indicators after glucose data collection)

**Supplementary Table S7.** Trends in the correlations between mean blood glucose and standard deviation of lag data for each physiological indicator (lag data for physiological indicators bofore glucose data collection)

**Supplementary Table S8.** Trends in the correlations between standard deviations of blood glucose values and standard deviations of lag data for each physiological indicator (lag data for physiological indicators after blood glucose data collection)

**Supplementary Table S9.** Results of the multiple regression analysis of the slope of the elevated blood glucose peak and the mean values of physiological and nutritional assessment indices.

**Supplementary Table S10.** Analysis of variance for the slopes of the peak of elevated blood glucose and the mean values of physiological indices and nutritional assessment indices

**Supplementary Table S11.** Results of the multiple regression analysis between the slope of the descending peak of blood glucose and the mean values of physiological and nutritional assessment indices.

**Supplementary Table S12.** Analysis of variance for the slope of the blood glucose descending peak and the mean values of physiological and nutritional assessment indices

**Supplementary Table S13.** Results of multiple regression analysis of the slope of the elevated blood glucose peak and SD of physiological indices.

**Supplementary Table S14.** Analysis of variance for the slope of the elevated blood glucose peak and standard deviation of physiological indices

**Supplementary Table S15.** Results of multiple regression analysis of the slope of the elevated blood glucose peak and SD of physiological indices.

**Supplementary Table S16.** Analysis of variance for the slope of the descending peak of blood glucose and standard deviation of physiological and nutritional assessment indices

**Supplementary Table S17.** Results of the multiple regression analysis of the slope of the elevated blood glucose peak and the mean values of physiological and nutritional assessment indices (Adjustment by sex).

**Supplementary Table S18.** Analysis of variance for the slope of the peak of elevated blood glucose and the mean values of physiological indices and nutritional assessment indices (Adjustment by sex)

**Supplementary Table S19.** Results of the multiple regression analysis between the slope of the descending peak of blood glucose and the mean values of physiological and nutritional assessment indices (Adjustment by sex)

**Supplementary Table S20.** Analysis of variance for the slope of the blood glucose descending peak and the mean values of physiological and nutritional assessment indices (Adjustment by sex)

**Supplementary Table 21.** Results of the multiple regression analysis of the slope of the elevated blood glucose peak and standard deviation of physiological indices (Adjustment by sex).

**Supplementary Table S22.** Analysis of variance for the slope of elevated blood glucose peak and standard deviation of physiological indices (Adjustment by sex)

**Supplementary Table S23.** Results of the multiple regression analysis between the slope of the descending peak of blood glucose and standard deviation of physiological and nutritional assessment indices (Adjustment by sex).

**Supplementary Table S24.** Analysis of variance for the slope of the descending peak of blood glucose and standard deviation of physiological and nutritional assessment indices (Adjustment by sex)

**Supplementary Table S25.** Relationship between the slope of blood glucose rise and the combination patterns (TEMP, ACC, HR, and EDA)

**Supplementary Table S26.** Summary of results

**Supplementary Table S27.** Relationship between the downward slope of blood glucose and the combination patterns (TEMP, ACC, HR, and EDA)

**Supplementary Table S28.** Summary of results

**Supplementary Table S29.** Relationship between the slope of the rise in blood glucose and dietary nutrient combination patterns (carbon, protein, calories, sugar, and fiber)

**Supplementary Table S30.** Summary of results

**Supplementary Table S31.** Relationship between the downward slope of blood glucose and the combination patterns (carbon, protein, calories, sugar, and fiber)

**Supplementary Table S32.** Summary of results

**Supplementary Table S33.** Summary of ANOVA results for groups with higher and lower than median blood glucose elevation and descent slopes and mean values of each physiological index and dietary nutrient values.

**Supplementary Figure S1.** Lag data before data collection time shift at eight time points (**−**120, **−**105, **−**90, **−**75, **−**60, **−**45, **−**30, and **−**15 min); ACC: tri-axial accelerometer derived acceleration.

**Supplementary Figure S2.** Lag data after data collection time shift at eight time points

(120, 105, 90, 75, 60, 45, 30, and 15 min).

**Supplementary Figure S3.** Trends in the correlations between mean blood glucose values and mean of lag data for each physiological indicator (lag data for physiological indicators after blood glucose data collection).

TEMP: skin temperature; ACC: tri-axial accelerometer derived acceleration; HR: heart rate; EDA: electrodermal activity

*: P-value of correlation coefficient P < 0.05

**Supplementary Figure S4.** Trends in the correlations between mean blood glucose values and lag data for each physiological indicator standard deviation (lag data for physiological indicators after blood glucose data collection).

TEMP: skin temperature; ACC: tri-axial accelerometer derived acceleration; HR: heart rate; EDA: electrodermal activity

*: P-value of correlation coefficient P < 0.05

**Supplementary Figure S5.** Trends in the correlations between standard deviations of blood glucose and standard deviations of lag data for each physiological indicator (lag data for physiological indicators before blood glucose data collection).

TEMP: skin temperature; ACC: tri-axial accelerometer derived acceleration; HR: heart rate; EDA: electrodermal activity

*: P-value of correlation coefficient P < 0.05

**Supplementary Figure S6.** Trends in the correlations between standard deviations of blood glucose values and standard deviations of lag data for each physiological indicator (lag data for physiological indicators after blood glucose data collection).

TEMP: skin temperature; ACC: tri-axial accelerometer derived acceleration; HR: heart rate; EDA: electrodermal activity

*: P-value of correlation coefficient P < 0.05

**Supplementary Figure S7.** Relationship between the downward slope of blood glucose and the combination patterns (skin temperature [TEMP], tri-axial accelerometer derived acceleration [ACC], heart rate [HR], and electrodermal activity [EDA]).

**Supplementary Figure S8** Relationship between the downward slope of blood glucose and the combination patterns (carbon, protein, calories, sugar, and fiber).

**Supplementary Table S1.** Studies using existing open data sets

|  | Article | Journal | Reference Database | No. of databases | Objective | Method |
| --- | --- | --- | --- | --- | --- | --- |
|  |  |  |  |  |  |  |
| 1 | Automated feature extraction from population wearable device data; identified novel loci associated with sleep and circadian rhythms [1] | PLoS Genetics | UK Biobank: an open access resource for identifying the causes of a wide range of complex diseases of middle and old age (2015) | 1 | Developed a computationally-efficient method for deriving the characteristics of circadian rhythms | Developed an unsupervised feature extraction method based on machine learning |
| 2 | Detected sleep outside the clinic using wearable heart rate devices [2] | Scientific Reports | Biobank validation study(2015)  Multi-ethnic study of atherosclerosis (2015)  PhysioNet Apple watch (2019)  PhysioNet Multilevel monitoring of activity and sleep in healthy people (2020) | 4 | Developed and tested heart rate-based algorithms that do not require human input | Developed and tested heart rate-based algorithms that do not require human input. Evaluated in four study cohorts using different research and consumer devices for over 2,000 nights |
| 3 | Artificial Intelligence-driven sleep staging from actigraphy and heart rate [3] | PLoS One | Multi-ethnic study of atherosclerosis (2015) MrOS Sleep Study (2011) PhysioNet Apple watch (2019) | 3 | Validated an artificial intelligence-based model for automated mobile sleep staging | Actigraphy and crude heart rate data from two independent study population cohorts were used to validate the model; a three-class staging model was extended to an unrelated Apple watch data set. |
| 4 | The combination of topological data analysis and mathematical modeling improved sleep stage prediction from consumer-grade wearables [4] | ﻿bioRxiv preprint | Multi-ethnic study of atherosclerosis (2015）  PhysioNet Apple watch (2019) | 2 | To develop a neural network algorithm to predict sleep stages. | To assess accuracy, the authors applied it to motion and heart rate data from an Apple watch worn by a participant undergoing polysomnography and compared the predicted sleep stages to the corresponding polysomnography ground truth recordings. |
| 5 | Personalized recognition of wake/sleep state based on the combined shapelets and K-means algorithm [5] | Biomed Signal Process Control | PhysioNet Apple watch (2019) PhysioNet Sleep apnea database (2010) PhysioNet multilevel monitoring of activity and sleep in healthy people (2020) | 3 | To propose an unsupervised method that requires only heart rate variability to recognize wake/sleep states to improve the generalization ability of wake/sleep recognition and avoid the negative effects of body movement, and validate the classification results in public. | The shapelet algorithm was used to quantify the similarity between heart rate variability segments, and the K-means clustering algorithm was used to improve the shapelet algorithm to achieve wake/sleep classification. Sleep tags in the database were used to validate and compare classification results before and after improvement. Analyzed the impact of unbalanced samples on the classification performance of the two algorithms. |
| 6 | Multitask deep learning for cost-effective prediction of patient’s length of stay and readmission state using multimodal physical activity sensory data [6] | IEEE J Biomed Heal Informatics | PhysioNet in-hospital physical activity measured using a new Bosch accelerometer sensor system (2020） | 1 | To develop a multimodal, multitasking long short-term memory deep learning model that can predict both length of stay and readmission for patients. | Multi-sensor data from 47 patients were divided into eight 1-h sections to construct time steps with six steps per hour. Using statistical features from this data, a model was developed to predict 30-day readmission as a binary classification and length of hospital stay as a regression task. |
| 7 | Impact of subject-specific training data on anxiety level classification from physiologic data [7] | 2021 IEEE International Symposium on Medical Measurements and Applications | PhysioNet electrocardiogram skin conductance and respiration from spider-fearing individuals watching spider video clips (2020) | 1 | To explore machine learning methods to predict two to three levels of anxiety when individuals with arachnophobia viewed spider images in controlled trials. | Features were extracted from electrocardiograms and skin electrical time series signals. Specifically, the authors explored the performance of the model as the amount of data associated with the test subject increased in the training set. They compared standard K-split cross-validation with leaky group split cross-validation with sample completion, systematically varying the number of test subject data included in the training set. |
| 8 | A neural network approach for anxiety detection based on ECG  [8] | 2021 9th E-Health Bioeng Conf EHB | PhysioNet electrocardiogram skin conductance and respiration from spider-fearing individuals watching spider video clips (2020) | 1 | To determine whether features computed with ultrashort heart rate variability may be used as a reliable measure to detect the onset of anxiety | A deep learning approach to detect anxiety based on very short-term heart rate variability measurements in arachnophobic individuals |
| 9 | Utility of the full ECG waveform for stress classification [9] | Sensors | PhysioNet electrocardiogram skin conductance and respiration from spider-fearing individuals watching spider video clips (2020) | 1 | To demonstrate the potential advantage of including small waves in binary stress classification as including heart rate variability features derived from the detection of small peaks (P, Q, S, T waves) in the electrocardiogram waveform is potentially advantageous. | Used an existing dataset of ECG recordings from 57 participants (ages 18-40) who self-reported fear of spiders when viewing the spider video; a model with only R-peak features was compared with two models with small peak features |
| 10 | Respiratory markers significantly enhance anxiety detection using multimodal physiological sensing [10] | BHI 2021 – 2021 IEEE EMBS Int Conf Biomed Heal Informatics, Proc | PhysioNet Electrocardiogram skin conductance and respiration from spider-fearing individuals watching spider video clips (2020) | 1 | While features extracted from electrocardiogram and electrodermal activity can be used to accurately distinguish between periods of phobic anxiety and periods of rest, here, the authors aimed to demonstrate that features extracted from respiratory effort signals can further enhance this capability. | Surveyed by adding respiration markers to the classifier and assessed the importance of features by sorting and deleting |
| 11 | Digital Health Data, a way to take under control the quality during the elaboration processes [11] | Proc - 16th Int Conf Signal-Image Technol Internet-Based Syst SITIS 2022 | PhysioBank PhysioToolkit and PhysioNet: Components of a new research resource for complex physiologic signals (2021） PPG-DaLiA Deep PPG: large-scale heart rate estimation with convolutional neural networks (2019) | 2 | To exclude unreliable events and obtain optimized portions of the data to compute appropriate statistics, evaluate data, and construct derived measurements | Investigated uncertainty in measured attributes of events by correlating them with other events occurring in a close time frame. Framework development occurred with several experiments using data from wearable devices (both synthetic data sets and publicly available benchmarks). |
| 12 | Detection of paroxysmal atrial fibrillation from dynamic ECG recordings based on a deep learning model [12] | J Pers Med; 13. Epub ahead of print | PhysioNet AF classification from a short single lead ECG recording (2017) PhysioNet paroxysmal atrial fibrillation events detection from dynamic ECG recordings: The 4th China physiological signal challenge (2021)  The impact of the MIT-BIH arrhythmia database. (2001)  The long-term AF database (LTAF) (2007)  The MIT-BIH atrial fibrillation database (AFDB)（1983） | 5 | To propose a new atrial fibrillation detection method for short-term electrocardiogram samples | Detected atrial fibrillation from the normal rhythm of the heart and its onset and termination by using a deep learning model to distinguish between atrial fibrillation and atrial flutter, both of which show similar patterns in the electrocardiogram. The proposed model uses a visual block and transformer encoder, data from the CPSC 2021 Challenge, and was tested on four public data sets to validate the usefulness of the proposed method. |
| 13 | A comparative study on neural networks for paroxysmal atrial fibrillation events detection from electrocardiography [13] | J Electrocardiol | PhysioNet paroxysmal atrial fibrillation events detection from dynamic ECG recordings: The 4th China physiological signal challenge (2021) | 1 | To conduct a comparative study of the impact of neural networks of different architectures on the detection of paroxysmal atrial fibrillation events from dynamic electrocardiogram recordings | A segmentation neural network model was used to predict atrial fibrillation at each sample point. |
| Studies 14–18 are diabetes-related studies | | | | | | |
| 14 | Deep physiological model for blood glucose prediction in T1DM patients [14] | Sensors (Switzerland） | The open D1NAMO dataset: A multi-modal dataset for research on non-invasive type 1 diabetes management (2018） The dataset was generated using the AIDA Diabetes Simulator (2020) | 2 | To review the state-of-the-art in predicting blood glucose in patients with type 1 diabetes and propose, implement, validate, and compare new hybrid models that decompose deep machine learning models to mimic the metabolic behavior of physiological glucose methods | Differential equations for carbohydrate and insulin absorption in the physiological model were modeled using a recurrent neural network implemented with a long short-term memory cell |
| 15 | Short-term prediction of future continuous glucose monitoring readings in type 1 diabetes: Development and validation of a neural network regression model [15] | Int J Med Inform | The open D1NAMO dataset: A multi-modal dataset for research on non-invasive type 1 diabetes management (2018）  REPLACE-BG: a randomized trial comparing continuous glucose monitoring with and without routine blood glucose monitoring in adults with well-controlled type 1 diabetes (2017） T1D Exchange Severe Hypoglycemia in Older adults with type 1 diabetes (2016） | 3 | To explore the possibility of developing and validating a model to predict future continuous glucose monitoring (CGM) measurements to overcome the time delay, which causes clinically significant differences between CGM and actual blood glucose levels, especially during rapid changes | An artificial neural network regression (NN) approach was used to predict continuous glucose monitoring (CGM) values with a 15-min lead time; the NN was trained and internally validated on 23 million min of CGM and externally validated on 2 million min of CGM. The validation included data from 278 patients with type 1 diabetes using three different CGM sensors; NN performance was compared with three alternative methods: linear extrapolation, spline extrapolation, and last observation carryover. |
| 16 | Ensemble methods in combination with compartment models for blood glucose level prediction in type 1 diabetes mellitus [16] | Comput Methods Programs Biomed | The open D1NAMO dataset: A multi-modal dataset for research on non-invasive type 1 diabetes management (2018） Blood glucose data collected from 16 patients in FN Motol University Hospital patients (2020) | 2 | To demonstrate the predictive ability of ensemble methods compared with individual algorithms while combining each method with compartmental models for simulating rapid-acting insulin absorption. | An approach combining the blood glucose prediction algorithms used was proposed, where three different ensemble methods (linear, bagging, and boosting metaregression) were applied to evaluate their ability to provide accurate predictions 30, 45, and 60 min into the forecast period. |
| 17 | Detection of physical activity using machine learning methods based on continuous blood glucose monitoring and heart rate signals [17] | Sensors | The Ohio T1DM dataset for blood glucose level prediction (2018) | 1 | To detect physical activity using wearable sensors that provide a continuous glucose monitoring system and heart rate | Established a simple machine learning-based algorithm using patient data to develop a physical activity detector |
| 18 | Machine-learning-based diabetes prediction using multi-sensor data  [18] | IEEE Sens J | The open D1NAMO dataset: A multi-modal dataset for research on non-invasive type 1 diabetes management (2018) | 1 | To predict diabetes using data from different types of wearables/sensors such as glucose, electrocardiogram, acceleration signals, and respiration sensors. To compare diabetes prediction results from individual sensor data with combined sensor data fused with features | Focused on the use of multiple sensor data to predict diabetes disease using machine learning algorithms and examined different combinations of health sensors to better predict diabetes. Which sensors and which combinations can predict diabetes disease more appropriately and with greater accuracy were also investigated. In addition, the optimal window size for diabetes prediction using different sensor data was evaluated. |

**Supplementary Table S2.** Outcomes of the search for a wearable-type device research database in PhysioNet.

| Database name | Author | Devices | Participants | End point |
| --- | --- | --- | --- | --- |
| CogWear: can we detect cognitive effort with consumer-grade wearables? | Michal K Grzeszczyk | - *Empatica E4*^a^ - *Samsung Galaxy Watch4* - Muse S EEG^b^ headband | - Pilot trials: 11 cases - Gaming trials: 13 cases | Detection of cognitively demanding task performance |
| SCG-RHC^c^: wearable seismocardiogram signal and right heart catheter database | Michael Chan | - ECG^d^ triaxial seismocardiogram | - 73 patients referred for hemodynamic evaluation of HF^e^ status | Assessment of patient’s clinical condition for HF |
| BIG IDEAs laboratory glycemic variability and wearable device data | Cho et al [29] | - Dexcom G6 CGM^f^ - *Empatica E4* | - 16 patients with prediabetic HbA_1c_^g^ levels between 5.3% and 6.4% | Generation of mobile health-based digital biomarkers of prediabetes and hyperglycemia risk |
| Wearable-based signals during physical exercises from patients with frailty after open-heart surgery | Daivaras Sokas | - Polar H10 | - 80 older patients with frailty who participated in a cardiac rehabilitation program after open heart surgery | Assessment of frail health status in a series of exercise tests |
| In-Gauge and En-Gage: understanding the occupants’ behavior, engagement, emotion, and comfort indoors with heterogeneous sensors and wearables | Nan Gao | - *Empatica E4* | - 23 student cases and 4 teacher cases | Examination of thermal comfort, engagement in learning, and emotions at school with daily surveys |
| Motion and heart rate from a wrist-worn wearable and labeled sleep from polysomnography | Olivia Walch | - *Apple Watch* | - 31 cases | Recording of raw acceleration and heart rate using an Apple watch while monitoring sleep via polysomnography |
| A wearable exam stress dataset for predicting cognitive performance in real-world settings | Md Rafiul Amin | - *Empatica E4* | - 10 cases | Measurement of the effect of stress on test performance |
| Electrocardiogram, skin conductance, and respiration from spider-fearing individuals watching spider video clips | Frank R Ihmig | - Wearable BITalino biosignal measurement device | - 57 cases of participants with arachnophobia | Detection of web anxiety levels from biometric signals |
| GLOBEM dataset: multiyear datasets for longitudinal human behavior modeling generalization | Xuhai Xu | - *Fitbit* | - 497 cases | Collection of data on longitudinal behavioral modeling physical activity and sleep behavior |
| GRABMyo^h^ | Ning Jiang | - EMGUSB2 - OT Bioelecttronica | - 43 healthy participants | EMG^i^-based gesture recognition research |
| BigIdeas_Lab_STEP: heart rate measurements captured by smartwatches for differing skin tones | Brinnae Bent | - *Apple Watch 4* - *Fitbit Charge 2* - *Garmin* - *Vivosmart 3* - *Xiaomi Miband 3* - *Empatica E4* - *Biovotion* - *Everion* | - 53 cases | Investigation of the accuracy of wearables and investigation of covariates such as skin color, signal lag, and device type |

^a^*Empatica E4 is a* wristband wearable devices.

^b^EEG: electroencephalogram.

^c^SCG-RHC: Seismocardiogram Signal and Right Heart Catheter

^d^ECG: electrocardiogram.

^e^HF: heart failure.

^f^CGM: continuous glucose monitor.

^g^HbA_1c_: glycated hemoglobin.

^h^GRABMyo: Gesture Recognition and Biometrics Electromyogram.

^i^EMG: electromyograph

**Supplementary Table S3.** Trends in the correlations between mean blood glucose and the mean of lag data for each physiological indicator (lag data for physiological indicators before blood glucose data collection)

| **Mean Glucose lag** | **Mean ACC** | | | **Mean HR** | | | **Mean TEMP** | | | **Mean EDA** | | | **Mean BVP** | | | **Mean IBI** | | |
| --- | --- | --- | --- | --- | --- | --- | --- | --- | --- | --- | --- | --- | --- | --- | --- | --- | --- | --- |
|  | N | R | *P* | N | R | *P* | N | R | *P* | N | R | *P* | N | R | *P* | N | R | *P* |
|  |  |  |  |  |  |  |  |  |  |  |  |  |  |  |  |  |  |  |
| **−120** | 9491 | 0.001 | .93 | 9491 | −0.068 | **<.001** | 9491 | 0.080 | **<.001** | 9069 | −0.115 | **<.001** | 9491 | 0.021 | **.04** | 5558 | 0.049 | **<.001** |
| **−105** | 9507 | 0.004 | .71 | 9507 | −0.076 | **<.001** | 9507 | 0.081 | **<.001** | 9084 | −0.119 | **<.001** | 9507 | 0.026 | **.01** | 5568 | 0.051 | **<.001** |
| **−90** | 9523 | 0.007 | .50 | 9523 | −0.084 | **<.001** | 9523 | 0.080 | **<.001** | 9099 | −0.127 | **<.001** | 9523 | 0.029 | **.005** | 5580 | 0.050 | **<.001** |
| **−75** | 9539 | 0.008 | .43 | 9539 | −0.088 | **<.001** | 9539 | 0.080 | **<.001** | 9114 | −0.133 | **<.001** | 9539 | 0.020 | **.045** | 5590 | 0.049 | **<.001** |
| **−60** | 9555 | 0.015 | .14 | 9555 | −0.097 | **<.001** | 9555 | 0.086 | **<.001** | 9129 | −0.138 | **<.001** | 9555 | 0.017 | .10 | 5601 | 0.051 | **<.001** |
| **−45** | 9571 | 0.022 | **.03** | 9571 | −0.109 | **<.001** | 9571 | 0.096 | **<.001** | 9144 | −0.141 | **<.001** | 9571 | 0.015 | .14 | 5610 | 0.052 | **<.001** |
| **−30** | 9587 | 0.019 | .07 | 9587 | −0.129 | **<.001** | 9587 | 0.111 | **<.001** | 9159 | −0.151 | **<.001** | 9587 | 0.016 | .11 | 5618 | 0.051 | **<.001** |
| **−15** | 9603 | 0.015 | .13 | 9603 | **−0.147** | **<.001** | 9603 | 0.126 | **<.001** | 9174 | **−0.164** | **<.001** | 9603 | 0.014 | .18 | 5624 | 0.048 | **<.001** |
| **0** | 9619 | 0.015 | .15 | 9619 | −0.143 | **<.001** | 9619 | **0.135** | **<.001** | 9189 | −0.161 | **<.001** | 9619 | 0.008 | .43 | 5629 | 0.049 | **<.001** |

N: number of data, R: correlation coefficient, P: p-value; ACC: tri-axial accelerometer derived acceleration; HR: heart rate; TEMP: skin temperature; BVP: blood volume pulse; EDA: electrodermal activity; IBI: inter-beat interval

**Supplementary Table S4.** Trends in the correlations between mean blood glucose and the mean of lag data for each physiological indicator mean (lag data for physiological indicators since blood glucose data collection)

| **Mean Glucose lag** | **Mean ACC** | | | **Mean HR** | | | **Mean TEMP** | | | **Mean EDA** | | | **Mean BVP** | | | **Mean IBI** | | |
| --- | --- | --- | --- | --- | --- | --- | --- | --- | --- | --- | --- | --- | --- | --- | --- | --- | --- | --- |
|  | N | R | *P* | N | R | *P* | N | R | *P* | N | R | *P* | N | R | *P* | N | R | *P* |
|  |  |  |  |  |  |  |  |  |  |  |  |  |  |  |  |  |  |  |
| **0** | 9619 | 0.015 | .15 | 9619 | −0.143 | **<.001** | 9619 | 0.135 | **<.001** | 9189 | **−0.161** | **<.001** | 9619 | 0.008 | .43 | 5629 | 0.049 | **<.001** |
| **15** | 9603 | 0.009 | .39 | 9603 | −0.133 | **<.001** | 9603 | 0.138 | **<.001** | 9173 | −0.151 | **<.001** | 9603 | 0.009 | .38 | 5622 | 0.053 | **<.001** |
| **30** | 9587 | 0.008 | .46 | 9587 | −0.140 | **<.001** | 9587 | 0.144 | **<.001** | 9157 | −0.142 | **<.001** | 9587 | 0.001 | .90 | 5617 | 0.061 | **<.001** |
| **45** | 9571 | 0.013 | .22 | 9571 | **−0.148** | **<.001** | 9571 | 0.155 | **<.001** | 9141 | −0.132 | **<.001** | 9571 | 0.005 | .64 | 5609 | 0.066 | **<.001** |
| **60** | 9555 | 0.026 | **.01** | 9555 | −0.139 | **<.001** | 9555 | **0.161** | **<.001** | 9125 | −0.123 | **<.001** | 9555 | 0.002 | .84 | 5601 | 0.073 | **<.001** |
| **75** | 9539 | 0.035 | **.001** | 9539 | −0.124 | **<.001** | 9539 | 0.157 | **<.001** | 9109 | −0.108 | **<.001** | 9539 | 0.002 | .82 | 5593 | 0.083 | **<.001** |
| **90** | 9523 | 0.029 | **.005** | 9523 | −0.120 | **<.001** | 9523 | 0.154 | **<.001** | 9093 | −0.092 | **<.001** | 9523 | 0.005 | .64 | 5588 | 0.098 | **<.001** |
| **105** | 9507 | 0.033 | **.001** | 9507 | −0.122 | **<.001** | 9507 | 0.153 | **<.001** | 9077 | −0.076 | **<.001** | 9507 | 0.002 | .88 | 5579 | 0.106 | **<.001** |
| **120** | 9491 | 0.033 | **.001** | 9491 | −0.121 | **<.001** | 9491 | 0.152 | **<.001** | 9061 | −0.062 | **<.001** | 9491 | 0.001 | .92 | 5573 | **0.120** | **<.001** |

N: number of data, R: correlation coefficient, P: p-value; ACC: tri-axial accelerometer derived acceleration; HR: heart rate; TEMP: skin temperature; BVP: blood volume pulse; EDA: electrodermal activity; IBI: inter-beat interval

**Supplementary Table S5.** Trends in the correlations between mean blood glucose and standard deviation of lag data for each physiological indicator (lag data for physiological indicators before blood glucose data collection)

| **Mean Glucose lag** | **SD ACC** | | | **SD HR** | | | **SD TEMP** | | | **SD EDA** | | | **SD BVP** | | | **SD IBI** | | |
| --- | --- | --- | --- | --- | --- | --- | --- | --- | --- | --- | --- | --- | --- | --- | --- | --- | --- | --- |
|  | N | R | *P* | N | R | *P* | N | R | P | N | R | *P* | N | R | P | N | R | *P* |
|  |  |  |  |  |  |  |  |  |  |  |  |  |  |  |  |  |  |  |
| **−120** | 8824 | −0.101 | **<.001** | 8824 | −0.055 | **<.001** | 8824 | −0.075 | **<.001** | 8401 | −0.170 | **<.001** | 8824 | −0.043 | **<.001** | 3370 | −0.021 | .23 |
| **−105** | 8839 | −0.105 | **<.001** | 8839 | −0.060 | **<.001** | 8839 | −0.075 | **<.001** | 8415 | −0.172 | **<.001** | 8839 | −0.040 | **<.001** | 3374 | −0.024 | .16 |
| **−90** | 8854 | −0.110 | **<.001** | 8854 | −0.065 | **<.001** | 8854 | −0.068 | **<.001** | 8429 | −0.182 | **<.001** | 8854 | −0.035 | **<.001** | 3379 | −0.025 | .14 |
| **−75** | 8869 | −0.112 | **<.001** | 8869 | −0.066 | **<.001** | 8869 | −0.066 | **<.001** | 8443 | −0.189 | **<.001** | 8869 | −0.029 | **.006** | 3383 | −0.023 | .19 |
| **−60** | 8884 | −0.120 | **<.001** | 8884 | −0.073 | **<.001** | 8884 | −0.074 | **<.001** | 8457 | −0.193 | **<.001** | 8884 | −0.028 | **.008** | 3390 | −0.026 | .13 |
| **−45** | 8899 | −0.136 | **<.001** | 8899 | −0.085 | **<.001** | 8899 | −0.087 | **<.001** | 8471 | −0.204 | **<.001** | 8899 | −0.038 | **<.001** | 3396 | −0.021 | .23 |
| **−30** | 8914 | −0.167 | **<.001** | 8914 | −0.105 | **<.001** | 8914 | −0.107 | **<.001** | 8485 | −0.223 | **<.001** | 8914 | −0.049 | **<.001** | 3403 | −0.016 | .35 |
| **−15** | 8929 | **−0.190** | **<.001** | 8929 | **−0.121** | **<.001** | 8929 | −0.120 | **<.001** | 8499 | **−0.237** | **<.001** | 8929 | −0.054 | **<.001** | 3407 | −0.024 | .16 |
| **0** | 8938 | −0.185 | **<.001** | 8938 | −0.117 | **<.001** | 8938 | **−0.121** | **<.001** | 8507 | −0.236 | **<.001** | 8938 | −0.049 | **<.001** | 3409 | −0.020 | .24 |

N: number of data, R: correlation coefficient, P: p-value, SD: standard deviation; ACC: tri-axial accelerometer derived acceleration; HR: heart rate; TEMP: skin temperature; BVP: blood volume pulse; EDA: electrodermal activity; IBI: inter-beat interval

**Supplementary Table S6.** Trends in the correlations between mean blood glucose and standard deviation of lag data for each physiological indicator (lag data for physiological indicators after glucose data collection)

| **Mean Glucose  lag** | **SD ACC** | | | **SD HR** | | | **SD TEMP** | | | **SD EDA** | | | **SD BVP** | | | **SD IBI** | | |
| --- | --- | --- | --- | --- | --- | --- | --- | --- | --- | --- | --- | --- | --- | --- | --- | --- | --- | --- |
|  | N | R | *P* | N | R | *P* | N | R | *P* | N | R | *P* | N | R | *P* | N | R | *P* |
|  |  |  |  |  |  |  |  |  |  |  |  |  |  |  |  |  |  |  |
| **0** | 8938 | **−0.185** | **<.001** | 8938 | −0.117 | **<.001** | 8938 | **−0.121** | **<.001** | 8507 | **−0.236** | **<.001** | 8938 | −0.049 | **<.001** | 3409 | −0.020 | .24 |
| **15** | 8923 | −0.170 | **<.001** | 8923 | −0.111 | **<.001** | 8923 | −0.117 | **<.001** | 8492 | −0.229 | **<.001** | 8923 | −0.049 | **<.001** | 3405 | −0.025 | .15 |
| **30** | 8908 | −0.171 | **<.001** | 8908 | −0.120 | **<.001** | 8908 | −0.113 | **<.001** | 8477 | −0.222 | **<.001** | 8908 | −0.056 | **<.001** | 3403 | −0.038 | **.03** |
| **45** | 8893 | −0.182 | **<.001** | 8893 | **−0.127** | **<.001** | 8893 | −0.119 | **<.001** | 8462 | −0.213 | **<.001** | 8893 | −0.061 | **<.001** | 3401 | −0.035 | **.04** |
| **60** | 8878 | −0.171 | **<.001** | 8878 | −0.117 | **<.001** | 8878 | −0.116 | **<.001** | 8447 | −0.196 | **<.001** | 8878 | −0.059 | **<.001** | 3401 | −0.036 | **.04** |
| **75** | 8863 | −0.158 | **<.001** | 8863 | −0.108 | **<.001** | 8863 | −0.107 | **<.001** | 8432 | −0.177 | **<.001** | 8863 | −0.053 | **<.001** | 3397 | −0.030 | .08 |
| **90** | 8848 | −0.151 | **<.001** | 8848 | −0.106 | **<.001** | 8848 | −0.094 | **<.001** | 8417 | −0.160 | **<.001** | 8848 | −0.047 | **<.001** | 3393 | −0.029 | .09 |
| **105** | 8833 | −0.141 | **<.001** | 8833 | −0.104 | **<.001** | 8833 | −0.084 | **<.001** | 8402 | −0.144 | **<.001** | 8833 | −0.043 | **<.001** | 3390 | −0.036 | **.04** |
| **120** | 8818 | −0.132 | **<.001** | 8818 | −0.096 | **<.001** | 8818 | −0.078 | **<.001** | 8387 | −0.131 | **<.001** | 8818 | −0.039 | **<.001** | 3386 | −0.032 | .06 |

N: number of data, R: correlation coefficient, P: p-value, SD: standard deviation; ACC: tri-axial accelerometer derived acceleration; HR: heart rate; TEMP: skin temperature BVP: blood volume pulse; EDA: electrodermal activity; IBI: inter-beat interval

**Supplementary Table S7.** Trends in the correlations between standard deviations of blood glucose values and standard deviations of lag data for each physiological indicator. (lag data for physiological indicators before blood glucose data collection)

| **SD Glucose lag** | **SD ACC** | | | **SD HR** | | | **SD TEMP** | | | **SD EDA** | | | **SD BVP** | | | **SD IBI** | | |
| --- | --- | --- | --- | --- | --- | --- | --- | --- | --- | --- | --- | --- | --- | --- | --- | --- | --- | --- |
|  | N | R | P | N | R | P | N | R | P | N | R | P | N | R | P | N | R | P |
|  |  |  |  |  |  |  |  |  |  |  |  |  |  |  |  |  |  |  |
| **−120** | 8818 | 0.112 | **<.001** | 8818 | 0.081 | **<.001** | 8818 | 0.038 | **<.001** | 8395 | 0.032 | **.003** | 8818 | −0.031 | **.003** | 3369 | 0.003 | .88 |
| **−105** | 8833 | 0.140 | **<.001** | 8833 | 0.096 | **<.001** | 8833 | 0.049 | **<.001** | 8409 | 0.044 | **<.001** | 8833 | −0.021 | **.049** | 3373 | 0.005 | .76 |
| **−90** | 8848 | 0.137 | **<.001** | 8848 | 0.109 | **<.001** | 8848 | 0.067 | **<.001** | 8423 | 0.048 | **<.001** | 8848 | −0.025 | **.018** | 3377 | −0.019 | .28 |
| **−75** | 8863 | 0.143 | **<.001** | 8863 | 0.101 | **<.001** | 8863 | 0.080 | **<.001** | 8437 | 0.039 | **<.001** | 8863 | −0.008 | .43 | 3382 | −0.039 | **.03** |
| **−60** | 8878 | 0.152 | **<.001** | 8878 | 0.112 | **<.001** | 8878 | 0.089 | **<.001** | 8451 | 0.030 | **.005** | 8878 | −0.012 | .24 | 3387 | −0.033 | **.05** |
| **−45** | 8893 | 0.149 | **<.001** | 8893 | 0.122 | **<.001** | 8893 | 0.103 | **<.001** | 8465 | 0.029 | **.007** | 8893 | −0.012 | .25 | 3393 | −0.039 | **.02** |
| **−30** | 8908 | 0.150 | **<.001** | 8908 | 0.102 | **<.001** | 8908 | 0.087 | **<.001** | 8479 | 0.021 | .06 | 8908 | −0.023 | **.03** | 3400 | −0.067 | **<.001** |
| **−15** | 8923 | 0.155 | **<.001** | 8923 | 0.132 | **<.001** | 8923 | 0.098 | **<.001** | 8493 | 0.013 | .25 | 8923 | −0.019 | .07 | 3406 | −0.039 | **.02** |
| **0** | 8938 | **0.157** | **<.001** | 8938 | **0.142** | **<.001** | 8938 | **0.127** | **<.001** | 8507 | 0.018 | .10 | 8938 | −0.017 | .11 | 3409 | −0.051 | **.003** |

N: number of data, R: correlation coefficient, P: p-value, SD: standard deviation; ACC: tri-axial accelerometer derived acceleration; HR: heart rate; TEMP: skin temperature; BVP: blood volume pulse; EDA: electrodermal activity; IBI: inter-beat interval

**Supplementary Table S8.** Trends in the correlations between standard deviations of blood glucose values and standard deviations of lag data for each physiological indicator (lag data for physiological indicators after blood glucose data collection)

| **SD Glucose**  **lag** | **SD ACC** | | | **SD HR** | | | **SD TEMP** | | | **SD EDA** | | | **SD BVP** | | | **SD IBI** | | |
| --- | --- | --- | --- | --- | --- | --- | --- | --- | --- | --- | --- | --- | --- | --- | --- | --- | --- | --- |
|  | N | R | *P* | N | R | *P* | N | R | *P* | N | R | *P* | N | R | *P* | N | R | *P* |
|  |  |  |  |  |  |  |  |  |  |  |  |  |  |  |  |  |  |  |
| **0** | 8938 | **0.157** | **<.001** | 8938 | **0.142** | **<.001** | 8938 | **0.127** | **<.001** | 8507 | 0.018 | .10 | 8938 | −0.017 | .10 | 3409 | −0.051 | **.003** |
| **15** | 8923 | 0.132 | **<.001** | 8923 | 0.100 | **<.001** | 8923 | 0.094 | **<.001** | 8492 | 0.000 | .96 | 8923 | −0.023 | **.03** | 3405 | −0.074 | **<.001** |
| **30** | 8908 | 0.125 | **<.001** | 8908 | 0.107 | **<.001** | 8908 | 0.079 | **<.001** | 8477 | 0.011 | .32 | 8908 | −0.034 | **.001** | 3403 | −0.057 | **.001** |
| **45** | 8893 | 0.126 | **<.001** | 8893 | 0.106 | **<.001** | 8893 | 0.085 | **<.001** | 8462 | 0.017 | .12 | 8893 | −0.025 | **.02** | 3401 | −0.066 | **<.001** |
| **60** | 8878 | 0.119 | **<.001** | 8878 | 0.101 | **<.001** | 8878 | 0.098 | **<.001** | 8447 | 0.001 | .90 | 8878 | −0.019 | .08 | 3401 | −0.080 | **<.001** |
| **75** | 8863 | 0.088 | **<.001** | 8863 | 0.073 | **<.001** | 8863 | 0.068 | **<.001** | 8432 | −0.010 | .35 | 8863 | −0.031 | **.003** | 3397 | −0.079 | **<.001** |
| **90** | 8848 | 0.086 | **<.001** | 8848 | 0.063 | **<.001** | 8848 | 0.058 | **<.001** | 8417 | −0.013 | .22 | 8848 | −0.023 | **.03** | 3393 | −0.100 | **<.001** |
| **105** | 8833 | 0.066 | **<.001** | 8833 | 0.051 | **<.001** | 8833 | 0.051 | **<.001** | 8402 | −0.001 | .94 | 8833 | −0.021 | .05 | 3390 | −0.067 | **<.001** |
| **120** | 8818 | 0.057 | **<.001** | 8818 | 0.033 | **<.001** | 8818 | 0.034 | **<.001** | 8387 | 0.000 | .98 | 8818 | −0.028 | **.009** | 3386 | −0.069 | **<.001** |

N: number of data, R: correlation coefficient, P: p-value, SD: standard deviation; ACC: tri-axial accelerometer derived acceleration; HR: heart rate; TEMP: skin temperature; BVP: blood volume pulse; EDA: electrodermal activity; IBI: inter-beat interval

**Supplementary Table S9.** Results of the multiple regression analysis of the slope of the elevated blood glucose peak and the mean values of physiological and nutritional assessment indices.

| Term | Estimate | SE | *t* test | *P* value |
| --- | --- | --- | --- | --- |
| Intercept | −0.475 | 0.495 | −0.96 | .34 |
| Mean (TEMP) ^a^ | 0.038 | 0.015 | 2.52 | **.01** |
| Mean (calorie) | −0.001 | 0.000 | −3.98 | **<.001** |
| Mean (carbon) | 0.010 | 0.001 | 6.53 | **<.001** |
| Mean (dietary fiber) | −0.020 | 0.008 | −2.51 | **.01** |
| Mean (protein) | 0.011 | 0.003 | 3.82 | **<.001** |

^a^TEMP: skin temperature

| Source | DF | Sum of Squares | Mean Square | F Ratio |
| --- | --- | --- | --- | --- |
|  |  |  |  |  |
| Model | 5 | 27.67 | 5.53 | 16.05 |
| Error | 457 | 157.53 | 0.34 | P−value |
| C.Total | 462 | 185.19 |  | **<.001** |

**Supplementary Table S10.** Analysis of variance for the slopes of the peak of elevated blood glucose and the mean values of physiological indices and nutritional assessment indices

**Supplementary Table S11.** Results of multiple regression analysis between the slope of the descending peak of blood glucose and the mean values of physiological and nutritional assessment indices.

| Term | Estimate | SE | *t* test | *P* value |
| --- | --- | --- | --- | --- |
| Intercept | −1.779 | 0.715 | −2.49 | .01 |
| Mean (ACC) | 0.029 | 0.011 | 2.67 | **.008** |
| Mean (HR_af)^a^ | −0.010 | 0.002 | −3.86 | **<.001** |
| Mean (HR_60)^b^ | −0.005 | 0.003 | −1.82 | .07 |
| Mean (HR_90)^c^ | 0.006 | 0.003 | 2.27 | **.02** |
| Mean (calorie) | 0.000 | 0.000 | 1.57 | .12 |
| Mean (sugar) | −0.006 | 0.002 | −3.72 | **<.001** |
| Mean (protein) | −0.003 | 0.002 | −1.35 | .18 |

^a^ACC: tri-axial accelerometer derived acceleration

^b^HR_af:: heart rate_af

^c^HR_60: heart rate_60

^d^HR_90: heart rate_90

**Supplementary Table S12.** Analysis of variance for the slope of the blood glucose descending peak and the mean values of physiological and nutritional assessment indices

| Source | DF | Sum of Squares | Mean Square | F Ratio |
| --- | --- | --- | --- | --- |
|  |  |  |  |  |
| Model | 7 | 10.34 | 1.48 | 5.88 |
| Error | 365 | 91.62 | 0.25 | P-value |
| C.Total | 372 | 101.96 |  | **<.001** |

**Supplementary Table S13.** Results of multiple regression analysis of the slope of the elevated blood glucose peak and SD of physiological indices.

| Term | Estimate | SE | *t* test | *P* value |
| --- | --- | --- | --- | --- |
| Intercept | 1.114 | 0.072 | 15.50 | **<.001** |
| SD (ACC)^a^ | −0.015 | 0.007 | −2.06 | **.04** |
| SD (HR_30)^b^ | −0.010 | 0.005 | −2.12 | **.035** |
| SD (TEMP_60)^c^ | −0.048 | 0.031 | −1.58 | .12 |
| SD (EDA_90)^d^ | 0.043 | 0.022 | 1.97 | **.049** |

^a^ACC: tri-axial accelerometer derived acceleration

^b^HR_30: heart rate_30

^c^TEMP_60: skin temperature_60

^d^EDA_90: electrodermal activity_90

**Supplementary Table S14.** Analysis of variance for the slope of the peak of elevated blood glucose and standard deviation of physiological indices

| Source | DF | Sum of Squares | Mean Square | F Ratio |
| --- | --- | --- | --- | --- |
|  |  |  |  |  |
| Model | 4 | 5.71 | 1.43 | 3.80 |
| Error | 413 | 154.94 | 0.38 | P-value |
| C.Total | 417 | 160.65 |  | **<.001** |

**Supplementary Table S15.** Results of the multiple regression analysis between the slope of the descending peak of blood glucose and SD of physiological and nutritional assessment indices.

| Term | Estimate | SE | *t* test | *P* value |
| --- | --- | --- | --- | --- |
| Intercept | −0.673 | 0.050 | −13.57 | <**.001** |
| SD (EDA_af)^a^ | 0.028 | 0.020 | 1.36 | .17 |
| SD (HR_60)^b^ | −0.005 | 0.004 | −1.42 | .16 |

^a^EDA_af: electrodermal activity_af

^b^HR_60: heart rate_60

**Supplementary Table S16.** Analysis of variance for the slope of the descending peak of blood glucose and standard deviation of physiological and nutritional assessment indices

| Source | DF | Sum of Squares | Mean Square | F Ratio |
| --- | --- | --- | --- | --- |
|  |  |  |  |  |
| Model | 2 | 1.01 | 0.503 | 1.89 |
| Error | 377 | 100.62 | 0.267 | P-value |
| C.Total | 379 | 101.63 |  | .15 |

**Supplementary Table S17.** Results of the multiple regression analysis of the slope of the elevated blood glucose peak and the mean values of physiological and nutritional assessment indices (Adjustment by sex)

| Term | Estimate | Std Error | t Ratio | *P-*value |
| --- | --- | --- | --- | --- |
|  |  |  |  |  |
| Intercept | -0.455 | 0.498 | -0.91 | .36 |
| Mean (TEMP)^a^ | 0.038 | 0.015 | 2.47 | **.014** |
| Mean (Calorie) | -0.001 | 0.000 | -3.96 | **<.001** |
| Mean (Carbon) | 0.010 | 0.001 | 6.53 | **<.001** |
| Mean (Dietary fiber) | -0.020 | 0.008 | -2.54 | **.01** |
| Mean (Protein) | 0.011 | 0.003 | 3.78 | **<.001** |
| sex[male] | -0.011 | 0.028 | -0.41 | .68 |

^a^TEMP: skin temperature

| Source | DF | Sum of Squares | Mean Square | F Ratio |
| --- | --- | --- | --- | --- |
|  |  |  |  |  |
| Model | 6 | 27.72 | 4.62 | 13.38 |
| Error | 456 | 157.47 | 0.35 | P-value |
| C.Total | 462 | 185.19 |  | **<.001** |

**Supplementary Table S18.** Analysis of variance for the slope of the peak of elevated blood glucose and the mean values of physiological indices and nutritional assessment indices (Adjustment by sex)

**Supplementary Table S19.** Results of the multiple regression analysis between the slope of the descending peak of blood glucose and the mean values of physiological and nutritional assessment indices (Adjustment by sex)

| Term | Estimate | Std Error | t Ratio | *P-*value |
| --- | --- | --- | --- | --- |
|  |  |  |  |  |
| Intercept | -1.783 | 0.720 | -2.48 | **.01** |
| Mean (ACC) | 0.029 | 0.011 | 2.64 | **.009** |
| Mean (HR_af) | -0.010 | 0.002 | -3.86 | **<.001** |
| Mean (HR_60) | -0.005 | 0.003 | -1.82 | .07 |
| Mean (HR_90) | 0.006 | 0.003 | 2.26 | **.03** |
| Mean (Calorie) | 0.000 | 0.000 | 1.57 | .12 |
| Mean (Sugar) | -0.006 | 0.002 | -3.69 | **<.001** |
| Mean (Protein) | -0.003 | 0.002 | -1.35 | .18 |
| sex[male] | -0.001 | 0.027 | -0.05 | .96 |

^a^ACC: tri-axial accelerometer derived acceleration

^b^HR_af:: heart rate_af

^c^HR_60: heart rate_60

^d^HR_90: heart rate_90

| Source | DF | Sum of Squares | Mean Square | F Ratio |
| --- | --- | --- | --- | --- |
|  |  |  |  |  |
| Model | 8 | 10.34 | 1.29 | 5.13 |
| Error | 364 | 91.62 | 0.25 | P-value |
| C.Total | 372 | 101.96 |  | **<.001** |

**Supplementary Table S20.** Analysis of variance for the slope of the blood glucose descending peak and the mean values of physiological and nutritional assessment indices (Adjustment by sex)

**Supplementary Table 21.** Results of the multiple regression analysis of the slope of the elevated blood glucose peak and standard deviation of physiological indices (Adjustment by sex)

| Term | Estimate | Std Error | t Ratio | *P­*-value |
| --- | --- | --- | --- | --- |
|  |  |  |  |  |
| Intercept | 1.107 | 0.073 | 15.25 | **<.001** |
| SD (ACC) ^a^ | -0.015 | 0.007 | -2.08 | **.04** |
| SD (HR_30)^b^ | -0.010 | 0.005 | -2.05 | **.04** |
| SD (TEMP_60) ^c^ | -0.047 | 0.031 | -1.54 | .12 |
| SD (EDA_90) ^d^ | 0.047 | 0.022 | 2.09 | .**04** |
| sex[male] | 0.022 | 0.031 | 0.71 | .48 |

^a^ACC: tri-axial accelerometer derived acceleration

^b^HR_30: heart rate_30

^c^TEMP_60: skin temperature_60

^d^EDA_90: electrodermal activity_90

**Supplementary Table S22.** Analysis of variance for the slope of the peak of elevated blood glucose and standard deviation of physiological indices (Adjustment by sex)

| Source | DF | Sum of Squares | Mean Square | F Ratio |
| --- | --- | --- | --- | --- |
|  |  |  |  |  |
| Model | 5 | 5.90 | 1.18 | 3.14 |
| Error | 412 | 154.75 | 0.38 | P-value |
| C.Total | 417 | 160.65 |  | **.009** |

**Supplementary Table S23.** Results of the multiple regression analysis between the slope of the descending peak of blood glucose and standard deviation of physiological and nutritional assessment indices (Adjustment by sex)

| Term | Estimate | Std Error | t Ratio | *P*-value |
| --- | --- | --- | --- | --- |
|  |  |  |  |  |
| Intercept | -0.673 | 0.050 | -13.35 | **<.001** |
| SD (EDA_af) ^a^ | 0.027 | 0.021 | 1.30 | .20 |
| SD (HR_60) ^b^ | -0.005 | 0.004 | -1.42 | .16 |
| sex[male] | -0.003 | 0.028 | -0.09 | .93 |

^a^EDA_af: electrodermal activity_af

^b^HR_60: heart rate_60

**Supplementary Table S24.** Analysis of variance for the slope of the descending peak of blood glucose and standard deviation of physiological and nutritional assessment indices (Adjustment by sex)

| Source | DF | Sum of Squares | Mean Square | F Ratio |
| --- | --- | --- | --- | --- |
|  |  |  |  |  |
| Model | 2 | 1.01 | 0.503 | 1.89 |
| Error | 377 | 100.62 | 0.267 | P-value |
| C.Total | 379 | 101.63 |  | .15 |

**Supplementary Table S25.** Relationship between the slope of blood glucose rise and the combination patterns (TEMP, ACC, HR, and EDA)

| Source | DF | Sum of Squares | Mean Square | F Ratio | *P­*-value |
| --- | --- | --- | --- | --- | --- |
|  |  |  |  |  |  |
| TEMP：ACC：HR：EDA | 15 | 8.54 | 0.57 | 1.48 | .11 |
| Error | 448 | 172.75 | 0.39 |  |  |
| C. Total | 463 | 181.29 |  |  |  |
|  |  |  |  |  |  |
| TEMP：ACC：HR： EDA | N | Mean | Std Error | Lower 95% | Upper 95% |
| 0000 | 37 | 0.803 | 0.102 | 0.603 | 1.004 |
| 0001 | 25 | 0.920 | 0.124 | 0.676 | 1.164 |
| 0010 | 31 | 0.940 | 0.112 | 0.721 | 1.159 |
| 0011 | 19 | 0.946 | 0.142 | 0.666 | 1.226 |
| 0100 | 34 | 0.775 | 0.107 | 0.566 | 0.985 |
| 0101 | 14 | 1.110 | 0.166 | 0.784 | 1.436 |
| 0110 | 34 | 0.734 | 0.107 | 0.525 | 0.944 |
| 0111 | 41 | 0.971 | 0.097 | 0.780 | 1.161 |
| 1000 | 35 | 1.140 | 0.105 | 0.934 | 1.347 |
| 1001 | 40 | 0.916 | 0.098 | 0.723 | 1.109 |
| 1010 | 15 | 0.975 | 0.160 | 0.660 | 1.290 |
| 1011 | 27 | 1.048 | 0.120 | 0.813 | 1.283 |
| 1100 | 24 | 0.969 | 0.127 | 0.720 | 1.218 |
| 1101 | 24 | 1.303 | 0.127 | 1.054 | 1.552 |
| 1110 | 23 | 0.876 | 0.129 | 0.621 | 1.130 |
| 1111 | 41 | 0.922 | 0.097 | 0.731 | 1.113 |

ACC: tri-axial accelerometer derived acceleration; HR: heart rate; TEMP: skin temperature; EDA: electrodermal activity

**Supplementary Table S26.** Summary of results

| TEMP：ACC：HR： EDA | N | Mean | Large or small |
| --- | --- | --- | --- |
|  |  |  |  |
| 0000 | 37 | 0.803 | Reference |
| 0001 | 25 | 0.920 | Slightly large |
| 0010 | 31 | 0.940 | Slightly large |
| 0011 | 19 | 0.946 | Slightly large |
| 0111 | 41 | 0.971 | Slightly large |
| 1001 | 40 | 0.916 | Slightly large |
| 1010 | 15 | 0.975 | Slightly large |
| 1100 | 24 | 0.969 | Slightly large |
| 1111 | 41 | 0.922 | Slightly large |
| 0101 | 14 | 1.110 | Large |
| 1000 | 35 | 1.140 | Large |
| 1011 | 27 | 1.048 | Large |
| 1101 | 24 | 1.303 | Large |

ACC: tri-axial accelerometer derived acceleration; HR: heart rate; TEMP: skin temperature; EDA: electrodermal activity

**Supplementary Table S27.** Relationship between the downward slope of blood glucose and the combination patterns (TEMP, ACC, HR, and EDA)

| Source | DF | Sum of Squares | Mean Square | F Ratio | *P*-value |
| --- | --- | --- | --- | --- | --- |
|  |  |  |  |  |  |
| TEMP：ACC：HR：EDA | 15 | 4.24 | 0.28 | 1.02 | .43 |
| Error | 394 | 109.24 | 0.28 |  |  |
| C. Total | 409 | 113.47 |  |  |  |
|  |  |  |  |  |  |
| TEMP：ACC：HR： EDA | N | Mean | Std Error | Lower 95% | Upper 95% |
| 0000 | 33 | −0.663 | 0.092 | −0.843 | −0.483 |
| 0001 | 23 | −0.635 | 0.110 | −0.851 | −0.419 |
| 0010 | 30 | −0.672 | 0.096 | −0.861 | −0.483 |
| 0011 | 18 | −0.627 | 0.124 | −0.871 | −0.383 |
| 0100 | 29 | −0.707 | 0.098 | −0.899 | −0.515 |
| 0101 | 12 | −0.695 | 0.152 | −0.994 | −0.396 |
| 0110 | 29 | −0.588 | 0.098 | −0.780 | −0.396 |
| 0111 | 37 | −0.751 | 0.087 | −0.921 | −0.580 |
| 1000 | 33 | −0.752 | 0.092 | −0.932 | −0.572 |
| 1001 | 32 | −0.689 | 0.093 | −0.872 | −0.506 |
| 1010 | 8 | −0.785 | 0.186 | −1.151 | −0.419 |
| 1011 | 25 | −1.045 | 0.105 | −1.252 | −0.838 |
| 1100 | 24 | −0.787 | 0.107 | −0.998 | −0.575 |
| 1101 | 18 | −0.588 | 0.124 | −0.832 | −0.344 |
| 1110 | 20 | −0.713 | 0.118 | −0.944 | −0.481 |
| 1111 | 39 | −0.678 | 0.084 | −0.844 | −0.512 |

ACC: tri-axial accelerometer derived acceleration; HR: heart rate; TEMP: skin temperature; EDA: electrodermal activity

**Supplementary Table S28.** Summary of results

| TEMP：ACC：HR：EDA | N | Mean | Large or small |
| --- | --- | --- | --- |
|  |  |  |  |
| 0000 | 33 | −0.663 | Reference |
| 0111 | 37 | −0.751 | Slightly large |
| 1000 | 33 | −0.752 | Slightly large |
| 1010 | 8 | −0.785 | Slightly large |
| 1100 | 24 | −0.787 | Slightly large |
| 1110 | 20 | −0.713 | Slightly large |
| 1011 | 25 | −1.045 | Large |

ACC: tri-axial accelerometer derived acceleration; HR: heart rate; TEMP: skin temperature; EDA: electrodermal activity

**Supplementary Table S29.** Relationship between the slope of the rise in blood glucose and dietary nutrient combination patterns (carbon, protein, calories, sugar, and fiber)

| Source | DF | Sum of Squares | Mean Square | F Ratio | *P*-value |
| --- | --- | --- | --- | --- | --- |
|  |  |  |  |  |  |
| Carbon: Protein: Calorie: Sugar: Fiber | 16 | 49.61 | 3.10 | 10.01 | <.001 |
| Error | 414 | 128.27 | 0.31 |  |  |
| C. Total | 430 | 177.88 |  |  |  |
|  |  |  |  |  |  |
| Carbon: Protein: Calorie: Sugar: Fiber | N | Mean | Std Error | Lower 95% | Upper 95% |
| 00000 | 91 | 0.599 | 0.058 | 0.484 | 0.714 |
| 00001 | 19 | 0.793 | 0.128 | 0.542 | 1.044 |
| 00010 | 26 | 0.784 | 0.109 | 0.569 | 0.998 |
| 01000 | 9 | 0.751 | 0.186 | 0.386 | 1.116 |
| 01001 | 17 | 0.506 | 0.135 | 0.241 | 0.771 |
| 01010 | 11 | 1.296 | 0.168 | 0.966 | 1.626 |
| 01011 | 6 | 1.084 | 0.227 | 0.638 | 1.531 |
| 01100 | 16 | 0.528 | 0.139 | 0.255 | 0.802 |
| 01101 | 21 | 0.792 | 0.121 | 0.554 | 1.031 |
| 10010 | 40 | 1.675 | 0.088 | 1.502 | 1.848 |
| 10011 | 6 | 1.181 | 0.227 | 0.734 | 1.627 |
| 10110 | 20 | 1.132 | 0.124 | 0.887 | 1.376 |
| 10111 | 10 | 1.084 | 0.176 | 0.738 | 1.430 |
| 11100 | 6 | 1.168 | 0.227 | 0.721 | 1.614 |
| 11101 | 38 | 1.019 | 0.090 | 0.841 | 1.196 |
| 11110 | 11 | 1.389 | 0.168 | 1.059 | 1.719 |
| 11111 | 84 | 1.172 | 0.061 | 1.052 | 1.291 |

**Supplementary Table S30.** Summary of results

| Carbon: Protein: Calorie: Sugar: Fiber | N | Mean | Large or small |
| --- | --- | --- | --- |
|  |  |  |  |
| 00000 | 91 | 0.599 | Reference |
| 01001 | 17 | 0.506 | Slightly small |
| 01000 | 9 | 0.751 | Slightly large |
| 00010 | 26 | 0.784 | Slightly large |
| 01101 | 21 | 0.792 | Slightly large |
| 00001 | 19 | 0.793 | Slightly large |
| 11101 | 38 | 1.019 | Slightly large |
| 10111 | 10 | 1.084 | Slightly large |
| 01011 | 6 | 1.084 | Slightly large |
| 10110 | 20 | 1.132 | Slightly large |
| 11100 | 6 | 1.168 | Slightly large |
| 11111 | 84 | 1.172 | Slightly large |
| 10011 | 6 | 1.181 | Slightly large |
| 01010 | 11 | 1.296 | Large |
| 11110 | 11 | 1.389 | large |
| 10010 | 40 | 1.675 | large |

**Supplementary Table S31.** Relationship between the downward slope of blood glucose and the combination patterns (carbon, protein, calories, sugar, and fiber)

| Source | DF | Sum of Squares | Mean Square | F Ratio | *P*-value |
| --- | --- | --- | --- | --- | --- |
|  |  |  |  |  |  |
| Carbon: Protein: Calorie: Sugar: Fiber | 16 | 11.03179 | 0.689487 | 2.6295 | <.001 |
| Error | 364 | 95.44425 | 0.262209 |  |  |
| C. Total | 380 | 106.47604 |  |  |  |
|  |  |  |  |  |  |
| Carbon: Protein: Calorie: Sugar: Fiber | N | Mean | Std Error | Lower 95% | Upper 95% |
| 00000 | 79 | −0.525 | 0.058 | −0.639 | −0.412 |
| 00001 | 16 | −0.577 | 0.128 | −0.829 | −0.325 |
| 00010 | 21 | −0.793 | 0.112 | −1.013 | −0.574 |
| 01000 | 8 | −0.526 | 0.181 | −0.882 | −0.170 |
| 01001 | 18 | −0.616 | 0.121 | −0.854 | −0.379 |
| 01010 | 10 | −1.012 | 0.162 | −1.331 | −0.694 |
| 01011 | 6 | −0.626 | 0.209 | −1.038 | −0.215 |
| 01100 | 13 | −0.408 | 0.142 | −0.687 | −0.128 |
| 01101 | 17 | −0.575 | 0.124 | −0.819 | −0.331 |
| 10010 | 36 | −1.028 | 0.085 | −1.196 | −0.860 |
| 10011 | 5 | −0.884 | 0.229 | −1.334 | −0.434 |
| 10110 | 18 | −0.826 | 0.121 | −1.063 | −0.588 |
| 10111 | 8 | −0.978 | 0.181 | −1.334 | −0.622 |
| 11100 | 4 | −0.769 | 0.256 | −1.272 | −0.265 |
| 11101 | 30 | −0.762 | 0.093 | −0.946 | −0.578 |
| 11110 | 11 | −0.783 | 0.154 | −1.086 | −0.479 |
| 11111 | 81 | −0.769 | 0.057 | −0.881 | −0.657 |

**Supplementary Table S32.** Summary of results

| Carbon: Protein: Calorie: Sugar: Fiber | N | Mean | Large or small |
| --- | --- | --- | --- |
|  |  |  |  |
| 00000 | 79 | −0.525 | Reference |
| 01100 | 13 | −0.408 | Slightly small |
| 01011 | 6 | −0.626 | Slightly large |
| 11101 | 30 | −0.762 | Slightly large |
| 11100 | 4 | −0.769 | Slightly large |
| 11111 | 81 | −0.769 | Slightly large |
| 11110 | 11 | −0.783 | Slightly large |
| 00010 | 21 | −0.793 | Slightly large |
| 10110 | 18 | −0.826 | Slightly large |
| 10011 | 5 | −0.884 | Slightly large |
| 10111 | 8 | −0.978 | Large |
| 01010 | 10 | −1.012 | Large |

**Supplementary Table S33.** Summary ANOVA results for groups with higher and lower than median blood glucose elevation and descent slopes and mean values of each physiological index and dietary nutrient values.

|  | The elevated slope of glucose | The downward slope of glucose |
| --- | --- | --- |
| Results of 1-way analysis of variance between blood glucose slope and mean values of physiological indicators and combined patterns of nutritional assessment indicators | TEMP：ACC：HR：EDA   \| TEMP：ACC：HR：EDA \| N \| Mean \| Large or small \| \| \| \| --- \| --- \| --- \| --- \| --- \| --- \| \| 0000 \| 37 \| 0.803 \| Reference \| \| \| \| 0001 \| 25 \| 0.920 \| Slightly large \| \| \| \| 0010 \| 31 \| 0.940 \| Slightly large \| \| \| \| 0011 \| 19 \| 0.946 \| Slightly large \| \| \| \| 0111 \| 41 \| 0.971 \| Slightly large \| \| \| \| 1001 \| 40 \| 0.916 \| Slightly large \| \| \| \| 1010 \| 15 \| 0.975 \| Slightly large \| \| \| \| 1100 \| 24 \| 0.969 \| Slightly large \| \| \| \| 1111 \| 41 \| 0.922 \| \| Slightly large \| \| 0101 \| 14 \| 1.110 \| \| Large \| \| 1000 \| 35 \| 1.140 \| \| Large \| \| 1011 \| 27 \| 1.048 \| \| Large \| \| 1101 \| 24 \| 1.303 \| \| Large \|   Carbon: protein: calorie: sugar: fiber   \| Carbon: protein: calories: sugar: fiber \| N \| Mean \| Large or small \| \| --- \| --- \| --- \| --- \| \| 00000 \| 91 \| 0.599 \| Reference \| \| 01001 \| 17 \| 0.506 \| Slightly small \| \| 01000 \| 9 \| 0.751 \| Slightly large \| \| 00010 \| 26 \| 0.784 \| Slightly large \| \| 01101 \| 21 \| 0.792 \| Slightly large \| \| 00001 \| 19 \| 0.793 \| Slightly large \| \| 11101 \| 38 \| 1.019 \| Slightly large \| \| 10111 \| 10 \| 1.084 \| Slightly large \| \| 01011 \| 6 \| 1.084 \| Slightly large \| \| 10110 \| 20 \| 1.132 \| Slightly large \| \| 11100 \| 6 \| 1.168 \| Slightly large \| \| 11111 \| 84 \| 1.172 \| Slightly large \| \| 10011 \| 6 \| 1.181 \| Slightly large \| \| 01010 \| 11 \| 1.296 \| Large \| \| 11110 \| 11 \| 1.389 \| Large \| \| 10010 \| 40 \| 1.675 \| Large \| | TEMP：ACC：HR：EDA   \| TEMP：ACC：HR：EDA \| N \| Mean \| Large or small \| \| --- \| --- \| --- \| --- \| \| 0000 \| 33 \| −0.663 \| Reference \| \| 0111 \| 37 \| −0.751 \| Slightly large \| \| 1000 \| 33 \| −0.752 \| Slightly large \| \| 1010 \| 8 \| −0.785 \| Slightly large \| \| 1100 \| 24 \| −0.787 \| Slightly large \| \| 1110 \| 20 \| −0.713 \| Slightly large \| \| 1011 \| 25 \| −1.045 \| Large \|   Carbon: protein: calorie: sugar: fiber   \| Carbon: protein: calorie: sugar: fiber \| N \| Mean \| Large or small \| \| --- \| --- \| --- \| --- \| \| 00000 \| 79 \| −0.525 \| Reference \| \| 01100 \| 13 \| −0.408 \| Slightly small \| \| 01011 \| 6 \| −0.626 \| Slightly large \| \| 11101 \| 30 \| −0.762 \| Slightly large \| \| 11100 \| 4 \| −0.769 \| Slightly large \| \| 11111 \| 81 \| −0.769 \| Slightly large \| \| 11110 \| 11 \| −0.783 \| Slightly large \| \| 00010 \| 21 \| −0.793 \| Slightly large \| \| 10110 \| 18 \| −0.826 \| Slightly large \| \| 10011 \| 5 \| −0.884 \| Slightly large \| \| 10111 \| 8 \| −0.978 \| Large \| \| 01010 \| 10 \| −1.012 \| Large \| \| 10010 \| 36 \| −1.028 \| Large \| |

ACC: tri-axial accelerometer derived acceleration; HR: heart rate; TEMP: skin temperature; EDA: electrodermal activity
